# COVID‐19 and individual performance in global virtual teams: The role of self‐regulation and individual cultural value orientations

**DOI:** 10.1002/job.2671

**Published:** 2022-11-10

**Authors:** Christopher Schlaegel, Marjaana Gunkel, Vas Taras

**Affiliations:** ^1^ Otto von Guericke University Magdeburg Magdeburg Germany; ^2^ Free University of Bozen—Bolzano Bolzano Italy; ^3^ University of North Carolina at Greensboro Greensboro North Carolina USA

**Keywords:** ambient stress, collectivism, COVID‐19 pandemic, individual performance, long‐term orientation, self‐regulation, uncertainty avoidance

## Abstract

Since the onset of the COVID‐19 pandemic, global virtual teams (GVTs) have become increasingly important. Drawing on conservation of resources theory and self‐regulation theory, we examined the mechanism and process underlying individuals' performance in GVTs in this specific situation. We posit that the local severity of the pandemic has a negative effect on individuals' performance in GVTs and that self‐regulation functions as a coping mechanism in times of pandemic‐related ambient stress, reducing its negative effect on performance. We suggest that three cultural value orientations, that is, uncertainty avoidance, collectivism, and long‐term orientation, explain different levels of self‐regulation, which in turn moderates the relationship between the local severity of the pandemic and individual performance in GVTs. Based on a sample of 2727 individuals from 31 countries participating in an international business consulting project during the early stage of the unfolding pandemic, we show that (a) the local severity of the pandemic had a negative effect on individuals' performance, (b) the negative effect of the pandemic on performance is weaker for individuals with high self‐regulation, and (c) uncertainty avoidance and long‐term orientation are positively associated with self‐regulation, which mediates the moderating relationship between the cultural value orientations and the relationship between the COVID‐19 pandemic and individual performance in GVTs.

## 1 INTRODUCTION

A pandemic is a widespread outbreak of an infectious disease that usually affects a considerable proportion of the population in one or more regions across the globe. It leads not only to a public health crisis but also to social, economic, and political disruption, affecting individuals across sectors, industries, and contexts. Despite the wide range of potential adverse effects, we have a limited understanding of the impact of major epidemics and pandemics on the performance of individuals in the non‐health sector (Collings et al., 2021). Because of physical distancing measures implemented worldwide in response to the COVID‐19 pandemic, many organizations have shifted to teleworking (World Economic Forum, 2020). As most tasks in organizations are organized around teams (McDaniel & Salas, 2018), many individuals have had to switch to working in global virtual teams (GVTs) during the pandemic. GVTs refer to a group of geographically dispersed people who work together on a project and utilize communication and information technologies to communicate and coordinate their efforts to accomplish a joint organizational task (Martins et al., 2004). Although a growing number of studies have examined the determinants of performance in GVTs (e.g., Hertel et al., 2005; Jimenez et al., 2017), the theoretical and empirical inquiry aspects are still at an early stage of development (Jimenez et al., 2017), specifically when it concerns the role of the COVID‐19 pandemic in GVT performance (e.g., Caligiuri et al., 2020; Kniffin et al., 2021). Thus, a better understanding of the factors that determine the performance of individuals working in GVTs during this pandemic is important for theory and practice.

Although researchers uncovered various individual‐level factors that contribute to a team member's performance in GVTs—such as broad personality traits (Cogliser et al., 2012), narrow personality traits, for example, self‐efficacy (Hardin et al., 2007), and individual abilities, such as intercultural competence (e.g., Presbitero, 2020)—the GVT literature has mainly focused on internal challenges of GVTs (e.g., Adamovic, 2018; Hertel et al., 2005) and has largely been silent on the role of GVT members' characteristics when they face external adversity. While several studies have stressed the importance of self‐regulation in the context of GVTs (e.g., Forester et al., 2007; Glazer et al., 2012), GVT research has focused on self‐regulation in a leadership context (e.g., Adamovic, 2018). Thus, there is a theoretical and empirical paucity when it comes to understanding whether, how, and when specific individual characteristics, such as self‐regulation, enable GVT team members to cope with external adversity (Caligiuri et al., 2020; Kniffin et al., 2021). In addressing this gap, the central research questions of our study are whether an external adverse event, such as the COVID‐19 pandemic, influences individuals' performance in GVTs and whether individuals' self‐regulation is an effective coping strategy for resisting the adverse effects of such a highly disruptive experience.

Conservation of resources (COR) theory (Hobfoll, 1989, 2001) describes how stressful circumstances unfold and explains that stress occurs when an individual's resources are threatened with loss when resources are lost or when an individual invests significant resources (e.g., effort) without gaining key resources in return. COR theory explicitly addresses the stress that occurs when natural disasters, such as the COVID‐19 pandemic, significantly impact individuals' health, social, and economic status (Hobfoll, 2012). The theory also suggests that the relationship between the occurrence of a natural disaster and the stress of individuals as their behavior intensifies, because people strive to obtain, retain, and protect the resources they value. Furthermore, it suggests that while core values differ across cultures, the resources that people value (e.g., health, well‐being, family, and wealth) are universal. When a highly destructive and disruptive natural disaster affects work life, social life, and personal life, all these resources are challenged or even lost (Hobfoll, 2012).

Previous research indicates that natural disasters lead to stress, which in turn is negatively related to performance outcomes (e.g., Helton & Head, 2012; Norris & Uhl, 1993). Thus, for individuals, the threat of the COVID‐19 pandemic, specifically at its onset, the uncertainty of not knowing how severe it will become, how long it will last, and how severe the consequences will be, most likely results in substantial stress. While acute stress may enable individuals to mobilize their resources and enhance their performance, long‐term stress impairs attention and motivation, resulting in reduced performance (e.g., Hunter & Thatcher, 2007). The way a pandemic unfolds can be described as a natural disaster occurring in slow motion (Taylor, 2019), and thus, stress persists over a longer time and becomes ambient in the environment (Campbell, 1983). Related research from the onset of the pandemic indicates that COVID‐19 can be characterized as an ongoing stressor (Fu et al., 2021). Furthermore, although the COVID‐19 pandemic spread quickly around the globe, pandemics in general and specifically at their onset are characterized by a widely varying number of infections and deaths in different countries (Taylor, 2019) and, thus, different levels of pandemic‐related stress (e.g., Kowal et al., 2020).

**FIGURE 1:**
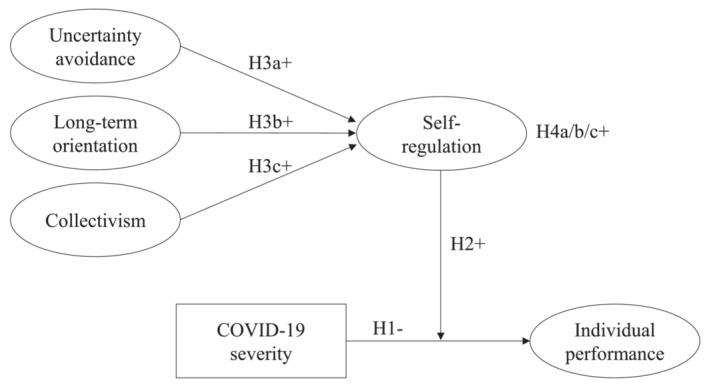
Conceptual model

Using COR theory as our overarching theoretical framework, we developed the conceptual model presented in Figure 1. We theorized that the consequences of the COVID‐19 pandemic include adverse effects on individuals' performance in GVTs. Specifically, we argue that ambient stress caused by the pandemic is likely to result in a reduction of energetic, cognitive, and emotional resources, resulting in decrements in performance. Although we do not directly assess individuals' perceived stress, we argue—based on existing theory and empirical evidence—that the local severity of the pandemic (assessed through the increase in the number of COVID‐19‐related deaths over a week) can be considered a reasonable proxy for the stress created by the pandemic. To pinpoint specific mechanisms involved in individual performance in GVTs, we drew from resource allocation models (e.g., Beal et al., 2005), which outline how the threat of resource loss influences the cognitive, affective, and behavioral aspects that contribute to individual performance.

Integrating the main tenets of COR theory and self‐regulation theory (Carver & Scheier, 1981), we argue that self‐regulation—the ability to control one's cognitions and behaviors—functions as a coping mechanism that reduces the negative effect of the pandemic on individual performance in GVTs. Specifically, we argue that self‐regulation guides individuals' use of affective and cognitive goal‐directed activities, which helps them cope with the adverse effects of the COVID‐19 pandemic. Finally, drawing on theories of cultural values (e.g., Hofstede, 1984; House et al., 2002) and the cultural‐embeddedness perspective of self‐regulation (e.g., Matsumoto et al., 2008; Trommsdorff & Rothbaum, 2008), we theorized and empirically tested the role of three individual cultural value orientations (i.e., uncertainty avoidance, long‐term orientation, and collectivism) as key antecedents of self‐regulation. Based on a large international sample of 2727 individuals from 31 countries, our study sheds light on the adverse performance effects of the pandemic and provides valuable insights into the cultural value orientations that determine self‐regulation and, therefore, into individuals' ability to manage the adverse effects caused by the pandemic.

Through the theoretical development and empirical testing of our conceptual model, we contribute to the literature in three ways. First, we contribute to GVT literature by examining the role of the pandemic on individual performance in GVTs. Although meta‐analytic evidence highlights the negative influence of stress on performance (Gilboa et al., 2008; LePine et al., 2005), we have only a limited understanding of the adverse effects of epidemics and pandemics on GVTs (Kniffin et al., 2021). Consistent with recent calls to examine the impact of the pandemic on relevant outcomes in GVTs (Caligiuri et al., 2020; Restubog et al., 2020), our study contributes to a better understanding of whether and to what extent the local severity of the pandemic affects individuals' performance in GVTs. In doing so, we extend prior theorizing on relevant antecedents of performance in GVTs, which focused on individual and team characteristics and only paid limited attention to environmental factors (Jimenez et al., 2017; Martins et al., 2004).

Second, following scholarly calls to combine COR theory with related theories to identify specific coping strategies (Hobfoll et al., 2018), we contribute to the literature on the role of such strategies for individual performance in GVTs (Degbey & Einola, 2020). While previous research (e.g., Mitchell et al., 2019) postulates that individuals' self‐regulation is a critical coping strategy in dealing with work stress and its facets (i.e., role stress and job insecurity), theoretically and empirically, little is known about the specific mechanisms through which self‐regulation enables individuals to cope with natural disasters in the context of task performance (Hobfoll et al., 2018). Our research highlights the role of self‐regulation as a coping strategy that reduces the adverse effects of stress caused by the pandemic. In doing so, we address calls to investigate such coping mechanisms in GVTs (Glazer et al., 2012) and extend existing theorizing on self‐regulation, which has focused on coping with leader‐, team‐, and organization‐related factors, but largely ignored the factors situated in the individuals' broader external environment. To this end, our study contributes to recent studies that have examined the determinants of performance in GVTs during the pandemic (e.g., Blanchard, 2021; Klonek et al., 2021; Whillans et al., 2021).

Third, geographic dispersion is an inherent characteristic of GVTs (Jimenez et al., 2017). Hence, the individuals working in GVTs are characterized by different cultural backgrounds and varying cultural value orientations. Previous studies have contributed to a better understanding of the role of cultural values in GVTs (e.g., Connaughton & Shuffler, 2007; Dekker et al., 2008; Shachaf, 2008; Staples & Zhao, 2006). In comparison to the GVT literature in general, this GVT research stream has mainly focused on the internal challenges of GVTs, and only a few studies have examined the cultural embeddedness of individual characteristics that are likely to enable the individual team members of GVTs to handle adversity caused by external factors (Hardin et al., 2007). Our pan‐cultural study contributes to the limited research that explains how coping strategies, such as self‐regulation, are related to individual cultural value orientations (e.g., Trommsdorff & Cole, 2011; Trommsdorff & Rothbaum, 2008). Globally and across the different waves, the COVID‐19 pandemic has influenced individuals regarding the fulfillment of their responsibilities, duties, and obligations. Coping strategies, such as self‐regulation, are rooted in individual cultural value orientations (Kurman, 2001; Morelli & Cunningham, 2012). Consequently, individuals who differ in these individual cultural value orientations may possess self‐regulation of varying degrees, and thus, they may differ in their ability to cope with the pandemic. We theoretically identify and empirically examine the effects of three individual cultural value orientations (i.e., uncertainty avoidance, long‐term orientation, and collectivism) that are positively associated with self‐regulation. By advancing this line of research, we extend theorizing on the antecedents of self‐regulation and contribute to the relatively scarce empirical research on the determinants of individuals' coping strategies (e.g., McCarthy et al., 2019; Wright et al., 2015). In doing so, we respond to recent calls to examine individuals' culturally linked responses to adverse events, such as pandemics (Cho, 2020; Guan et al., 2020), and unpack the individual cultural underpinnings of coping strategies in COR theory (e.g., Hobfoll et al., 2018).

## 2 THEORETICAL BACKGROUND AND HYPOTHESES

### 2.1 The influence of the COVID‐19 pandemic on individuals' performance in GVTs

The widespread effects of uncertainty and health‐related fears accompanying the COVID‐19 pandemic and the loss or disruption of daily routines, restricted social connections, and economic uncertainty are likely to cause stress (e.g., Fu et al., 2021; Taylor, 2019). The occurrence of the pandemic causes acute stress that, due to the long‐term horizon and the omnipresence of the pandemic in daily life, transforms into ambient stress. Such ambient stressors are “chronic, global conditions of the environment […] which, as stressors, place demand upon us to adapt or cope” (Campbell, 1983, p. 360). Ambient stressors are background conditions that influence individuals at nearly all levels, including affect, physiology, motivation, cognition, and behavior. They may even pass unnoticed unless they influence individuals' goals or threaten their health (e.g., Evans, 1984). Ambient stressors are unpleasant, rather long in duration, and intractable, thus creating adaptive challenges for individuals (e.g., Stokols, 1982). These stressors are of personal significance to everyone, depending on the need or desire to reduce the demands created by the stressor. The appraisal of ambient stressors is determined by contextual and personal factors that determine how vulnerable one is to the adverse effects of stressors (Campbell, 1983). Contextual factors refer to the enduring properties of *situations* and *settings* that mediate the relations between behavior and more transient environmental conditions (Stokols, 1982), such as duration of exposure, expected future events, personal resources (e.g., economic support, social support), or life‐choice context (e.g., family, job, residence). Personal factors such as age, health status, personality, and knowledge determine how vulnerable one is to the adverse effects of ambient stressors (Campbell, 1983). Thus, each individual experiences ambient stressors in a unique manner, depending on the impact of specific contextual and personal factors.

COR theory (Hobfoll, 2001, 2011) posits that the threat of resource loss is one of the main causes of stress, which in turn affects individual performance (e.g., Halbesleben & Bowler, 2007). Pandemics are associated with various stressors, including health‐related threats to oneself and to loved ones, severe disruptions of daily personal and work routines, loss of employment and income, and social isolation due to (self) quarantine and other social distancing measures leading to separation from family, friends, and colleagues (Taylor, 2019). COR theory argues that what people fundamentally value is universal across cultures and that natural disasters, such as major epidemics, “present objective elements of threat and loss” (Hobfoll, 2012, p. 228). Thus, according to COR theory, the social, economic, and psychological impact of a major epidemic—like the COVID‐19 pandemic—represents a severe threat to people around the globe (Monnier & Hobfoll, 2000). Furthermore, GVTs require their members to communicate across time zones and cultures, using online communication tools, which presents more challenges and threats of resource losses (e.g., Mesmer‐Magnus et al., 2011; Nurmi, 2011).

A fundamental assumption of resource allocation models is that individual performance is influenced by a combination of cognitive, affective, and behavioral aspects involving individual resources. These resources are limited (Beal et al., 2005). A global crisis that impacts nearly all aspects of life, such as the crisis caused by the COVID‐19 pandemic, influences our attention and thereby directs our feelings, thoughts, and actions toward the pandemic situation (e.g., Garfin et al., 2020). This has direct consequences for the presence and allocation of resources necessary for successful task performance. The cognitive resources required to carry out task‐related activities are likely to be reduced as members of a GVT, in their effort to reduce the threat of resource losses, use their cognitive resources to rapidly acquire, evaluate, and process pandemic‐related information, deal with unfamiliar situations, and solve previously unencountered problems (e.g., Hamilton et al., 2011). During a pandemic, widespread emotional stress occurs (Taylor, 2019) and the threat of resource loss results in negative emotions (Hobfoll, 1989, 2001). This distracts people's attention and disrupts efficient resource allocation, which adversely affects performance (Beal et al., 2005). Successful performance in GVTs requires that the cognitive and affective resources and the resource‐guided behavioral responses of the individuals working in these teams are directed toward the specific tasks necessary to achieve the desired outcome. However, the pandemic‐related threat of resource loss is likely to disrupt people's performance, as the ambient stress caused by the pandemic reduces the time, energy, and attention needed to fulfill tasks and achieve determined goals in the GVT. Different countries and regions are often at different stages of a pandemic, either in general or during a specific pandemic wave (Taylor, 2019). As a result, a pandemic has varying degrees of severity around the world, resulting in cross‐country differences in perceived stress (e.g., Kowal et al., 2020), thus allowing us to examine the influence of the pandemic on individuals' performance in GVTs. Therefore, we advance the notion that the local severity of the pandemic negatively influences individual performance in GVTs. The threat of resource losses caused by the pandemic disrupts the attention of individuals and makes it harder to allocate limited resources and continue with the activities necessary to pursue targets and achieve results in GVTs.

Hypothesis 1 COVID‐19 severity is negatively associated with individual performance in GVTs.

### 2.2 The moderating role of self‐regulation

Although researchers have proposed that various affective, cognitive, and behavioral strategies matter when dealing with stress, theoretically and empirically, little is known about the specific mechanisms through which these strategies enable individuals to cope with natural disasters in the context of task performance (Hobfoll et al., 2018). Self‐regulation theory suggests that individuals actively manage their performance and use self‐regulation to guide their goal‐directed activities and performance by setting their own standards and monitoring their progress toward these standards (Bandura, 1991; Carver & Scheier, 1981). Although meta‐analytic evidence shows that self‐regulation is positively associated with individuals' work performance in general (Cellar et al., 2011), we still have a limited understanding of the role of self‐regulation in individuals' task performance in GVTs (Jimenez et al., 2017).

Self‐regulation includes affective, cognitive, and behavioral components as it involves focusing and maintaining attention, initiating or inhibiting actions, thoughts, and emotions, as well as monitoring the results to achieve a particular goal (McClelland & Cameron, 2012). Emotional self‐regulation refers to the processes that serve the function of adapting emotional experiences and expressions, as well as the function of monitoring, evaluating, and modifying the intensity and duration of emotions to accomplish goals, to which individuals should apply social rules and standards of behavior (Kanfer, 2005; Wood, 2005). Cognitive self‐regulation refers to the ability to inhibit dominant or automatic responses and to flexibly shift the focus of attention (Boekaerts et al., 2005). Behavioral self‐regulation refers to the conscious and unconscious management and control of behaviors for the purpose of goal attainment (Carver & Scheier, 2000), that is, engaging (or not engaging) in behavior to achieve a goal. Together, the three self‐regulation facets enable individuals to emotions, thoughts, and actions to a broad range of situations (Baumeister & Vohs, 2007).

Drawing on theories of self‐regulation (Bandura, 1991; Carver & Scheier, 1981), our general theoretical argument is that individuals who possess more self‐regulation are more effective in inhibiting and controlling inappropriate responses that may arise because of the ambient stress caused by the pandemic when working in GVTs. Virtual work entails less monitoring by peers, which gives individuals more discretion on when, how, and under which conditions they complete their tasks, thereby increasing the risk of task avoidance and other negative task‐related attitudes and behaviors (Felstead et al., 2003). Members of GVTs receive less or no immediate feedback and guidance, which not only increases the chance of errors, but can be costly to correct at a later stage (Allen et al., 2015). Thus, more personal behavioral and cognitive self‐regulation in the form of self‐observation, self‐goal‐setting, and self‐rewarding are required (Bandura, 1991; Keith & Frese, 2005; Müller & Niessen, 2019), by limiting non‐work‐related internet surfing during the time devoted to task completion and by avoiding other distractions. Individuals working in GVTs, for example, can avoid strong reactive behaviors as an adaptive response to the abnormal pandemic situation (e.g., excessive news consumption) by proactively building daily schedules that follow a consistent and familiar structure, and that allow them to focus on the behaviors necessary to fulfill the tasks required to achieve the GVT goal. Furthermore, when problems with GVT members arise, emotional and cognitive self‐regulation limits inappropriate behavioral responses to negative emotions and to refocuses attention on the completion of tasks needed to achieve the GVT goals (de Jonge & Dormann, 2006). For example, individuals working in GVTs can, by various means (e.g., small group video calls and one‐on‐one video calls), proactively maintain communication with team members to avoid misunderstandings and negative emotions.

A central principle of COR, namely, that individuals employ key psychological resources to regulate themselves to retain and protect valued resources (Hobfoll, 2012), allows us to combine COR theory and theories related to self‐regulation (Bandura, 1991; Carver & Scheier, 2011). It also allows us to identify self‐regulation as a coping mechanism that reduces the pandemic's negative consequences. Confronted with a problem situation, workers must set specific goals and plans and monitor their performance to reduce discrepancies between the individual's current state and the desired state while handling the situation (Carver & Scheier, 2011). Self‐regulation helps team members to cope with pandemic‐related factors, such as attention disruptions, rapid changes, and challenges caused by cognitive, affective, and behavioral mechanisms. In highly dynamic environments, cognitive self‐regulation allows individuals to respond to changing circumstances and to maintain desired performance outcomes by redirecting their attention to goal‐relevant information and activities (Kanfer & Ackerman, 1989; Tsui & Ashford, 1994). This means that instead of searching for and processing information related to the pandemic, employees ignore these distractions and actively direct their cognitive resources toward work‐related problems. Emotional self‐regulation helps team members to control the type, timing, and expression of their emotions (Gross & Thompson, 2007). Emotional self‐regulation enables individuals to reduce distraction and to redirect their attention from strong pandemic‐related emotions, such as extensive fear (e.g., fear for their health, family, and job), anxiety (e.g., anxiety related to a life threat), and sadness (e.g., sadness related to social distancing and isolation), to work‐related aspects that meet goal‐related requirements. Furthermore, emotional self‐regulation enables people to regulate the intensity of their affective response (e.g., not erupting in anger in a video call when confronted with technological problems), which is beneficial, especially in the challenging situation at the onset of a pandemic (e.g., Restubog et al., 2020). People with high levels of behavioral self‐regulation are better able to apply standards as guidelines for their behavior, enabling them to adapt successfully to environmental changes and demands (e.g., follow new rules, pay attention to new instructions) and, thus, to be more effective in different contexts (e.g., Vohs & Baumeister, 2004). This makes self‐regulation a protective mechanism that prevents the waste of limited resources, such as spending too much time on pandemic‐related thoughts, feelings, and actions that are not beneficial for attaining GVT‐related goals and objectives. Thus:

Hypothesis 2 The negative relationship between COVID‐19 severity and individual performance in GVTs is weaker for individuals with more self‐regulation than for those with less self‐regulation.

### 2.3 Individual cultural value orientations, self‐regulation, and individual performance

The COVID‐19 pandemic is a global crisis that challenges and disrupts the daily life and work practices of individuals worldwide. Given the growing use of GVTs in general, and specifically during the COVID‐19 pandemic, a crucial factor to consider when examining individuals' performance in GVTs is the cultural background of the geographically dispersed team members. Drawing on a cultural‐embeddedness perspective of self‐regulation (e.g., Matsumoto et al., 2008; Trommsdorff & Rothbaum, 2008), we argue that cultural values determine individuals' self‐regulation. Hence, cultural values indirectly influence the performance of GVTs through the moderating effect of self‐regulation on the relationship between the pandemic and individuals' performance. Cultural values are situated in a temporal, spatial, and social context (Oyserman & Lee, 2007). Thus, researchers differentiate between individual cultural value orientations and the cultural values of a society (Leung, 1989). Cultural values at the societal level refer to the widely shared ideas of a group about what is good, right, and desirable, and which guide the actions and evaluations of individuals in this society (Hofstede, 2001). Thus, cultural values provide a framework within which individual actions take place. Individual cultural value orientations reflect the strength of an individual's belief in the fundamental cultural values of society (Triandis, 1995).

Cultural values are intergenerationally transferred via the socialization of individuals in society. The socialization process is intricately linked to the motivation to act according to society's expectations. These expectations, in turn, are influenced by cultural values (Trommsdorff, 2009). When individuals try to meet the expectations of relevant others (e.g., parents, friends, close relatives, and role models), they internalize the dominant cultural values in society. Accordingly, in the socialization process, the development of self‐regulation in an individual is characterized by the efforts of the individual to modify internal affective and cognitive processes as well as behaviors to achieve specific goals and to fulfill the expectations of significant others who are situated in a cultural context that prioritizes specific cultural values. Consequently, individuals develop self‐regulation abilities that fit the set of individual cultural value orientations they developed within the society to which they belong (Trommsdorff, 2009). Thus, individual cultural value orientations influence how individuals learn to self‐regulate, form self‐regulation, and set goals of self‐regulation. As a result, in a situation where individuals who are located in different regions of the world are supposed to self‐regulate (e.g., GVT members during a pandemic), individual cultural value orientations are likely to influence self‐regulation and, therewith, the ability to cope with adverse effects. This cultural‐embeddedness perspective of self‐regulation allows us to better understand the differences in self‐regulation coping strategies among individuals from different cultural backgrounds (i.e., variations across countries). In addition, due to individual cultural value orientations (i.e., variations within countries), it helps us to understand the uniqueness of individual development within a given cultural environment. When applied to the role of self‐regulation in GVT performance during the pandemic, individuals from diverse cultural backgrounds and with different individual cultural value orientations are likely to have different self‐regulatory coping strategies to handle the pandemic's adverse effects. In the next paragraphs, we identify three specific cultural values that are important in the development of self‐regulation.

Drawing on the cultural value dimensions singled out by Hofstede (2001), we argue that uncertainty avoidance, long‐term orientation, and collectivism are particularly relevant in the development of individuals' self‐regulation (Bateman & Barry, 2012; Roney & Sorrentino, 1995; Trommsdorff, 2009). 1 Uncertainty is an inherent characteristic of a pandemic (Taylor, 2019). Most, if not all people experience uncertainty regarding the COVID‐19 pandemic's personal, social, and economic consequences. The way that individuals manage uncertainty varies considerably across countries. At a societal level, uncertainty avoidance refers to the tolerance of ambiguity and uncertainty and the “level of stress in a society in the face of an unknown future” (Hofstede, 2001, p. 29). At an individual level, uncertainty avoidance refers to the extent to which individuals avoid unfamiliar situations by preferring clear rules, routines, and consistency that provide a more predictable and controllable environment (Shuper et al., 2004). As a cultural antecedent of self‐regulation, uncertainty avoidance shows an inhomogeneous pattern across the three facets of self‐regulation. Focusing on affective self‐regulation, previous research suggests that individuals with a low uncertainty avoidance orientation show relatively high levels of emotional regulation and experience lower levels of anxiety and stress. Conversely, individuals with high uncertainty avoidance show relatively low levels of emotional regulation and higher levels of anxiety and stress when confronted with uncertainty (Hofstede, 1984, 2001).

In contrast to affective self‐regulation, previous studies on cognitive and behavioral self‐regulation argue that individuals who score high on uncertainty avoidance show high self‐regulation. This is because they have been socialized to manage uncertainty through a stronger emphasis of more self‐reliant behavior as they try to overcome challenges on their way to the desired goal (Kumar et al., 2019). Uncertainty implies that individuals have an incomplete description of the world and that they have less control over unstructured, unclear, and unpredictable uncertain situations. Individuals high in uncertainty avoidance are socialized to avoid such situations, and they prefer highly structured situations and stick to existing routines (Hofstede, 2001). At the onset of a pandemic, it is not possible or at least unlikely for most people to avoid this uncertainty, so individuals are confronted with uncertainty in a wide variety of forms. When individuals who usually try to avoid uncertainty are obliged to face it, they are socialized to manage this unstructured and unpredictable situation by controlling what they can control—their thoughts and behaviors (Roney & Sorrentino, 1995). Thus, individuals high in uncertainty avoidance strive to return to more structured situations by self‐regulation, among others, by following structured daily routines, leaving few ambiguities, and redirecting their thoughts toward a goal. By maintaining relevant codes of conduct, standards of practice, and rituals, individuals create a more favorable, stable, and predictable task environment (e.g., maintaining a structured daily routine). Consequently, individuals with a high uncertainty avoidance orientation will show higher levels of cognitive and behavioral self‐regulation, as these higher levels enable them to achieve a general sense of control by overcoming initial impulses and responses to uncertainty (Roney & Sorrentino, 1995; Sorrentino et al., 2003). Overall, while individuals with a high uncertainty avoidance orientation should have less affective self‐regulation, they should also have more cognitive and behavioral self‐regulation, resulting in a positive relationship between their uncertainty avoidance orientations and self‐regulation.

At a societal level, long‐term orientation refers to a society's orientation toward future rewards and “the choice of focus for people's efforts: the future or the present” (Hofstede, 2001, p. 29). At an individual level, long‐term orientation refers to an individual's holistic view of time, “… valuing both the past and the future rather than deeming actions important only for their effects in the here and now or the short term” (Bearden et al., 2006, p. 457). Individuals high in long‐term orientation are socialized to value planning, hard work for future benefits, and perseverance. Long‐term orientation can be considered motivational, as the current situation is evaluated and interpreted concerning possible future states. These possible states also represent specific hopes and fears that motivate the pursuit or avoidance of specific behaviors. As self‐regulation guides an individual's resources and activities over time and across changing situations, self‐regulation is important for directing feelings, thoughts, and behavior over a longer period (Bandura, 1988, 1991). This suggests that affective, cognitive, and behavioral self‐regulatory resources should be evident in individuals who value long‐term goals (Bateman & Barry, 2012). For instance, a salient representation of the self in the future as a team member who contributes less to the team efforts and who shows lower performance motivates self‐regulated behavioral choices and actions that do not remove time and attention from working on the team project. For example, individuals who score high on long‐term orientation, due to their focus on long‐term work‐related goals, use their self‐regulation to direct their attention to work‐related tasks. They do not constantly monitor pandemic‐related news or disrupt their workflow and work routines. In summary, individuals with a higher long‐term orientation are likely to have more self‐regulation.

At a societal level, individualism and collectivism appear as opposite poles of a continuum and reflect “… the relationship between the individual and the collectivity which prevails in a given society …” (Hofstede, 1984, p. 148). Here, individualism is reflected in laws and norms that encourage individuality, individual freedom, and a preference for individual work. Collectivism, by contrast, is characterized by a tight social framework in which people distinguish between in‐groups and out‐groups, with members identifying themselves in terms of their membership (Hofstede, 2001). At an individual level, collectivism is characterized by the ability to accept the norms of an “in‐group,” understand its hierarchy, share its resources, and consider the implications of actions concerning it (Hui & Triandis, 1986). The self‐regulation of individuals with collectivistic cultural value orientations is embedded in a socialization‐induced goal to give something back to the in‐groups (Trommsdorff & Rothbaum, 2008). Conversely, for an individual with an individualistic cultural value orientation, self‐regulation is rooted in the emotional experiences of pride and deservingness (Trommsdorff, 2009). Collectivistic cultures value socially engaged emotions such as gratitude, empathy, and shame more than individualistic cultures (Kitayama, 2000). As failure (e.g., not reaching certain goals) may lead to shame, individuals with a collectivistic cultural value orientation are assumed to show self‐regulation to avoid any resulting shame, which may extend to the entire family or work team (Trommsdorff, 2009). Given the different focuses of cultural value orientations, we argue that when working on a group project, it can be assumed that individuals with collectivistic cultural value orientations will exhibit higher levels of self‐regulation to attain the group's common goals and avoid shame.

Hypothesis 3 Uncertainty avoidance (H3a), long‐term orientation (H3b), and collectivism (H3c) are positively associated with self‐regulation.

Hypothesis 2 predicts that self‐regulation moderates the negative relationship between COVID‐19 severity and individual performance. Hypotheses 3a and 3b predict a positive relationship between uncertainty avoidance, long‐term orientation, and self‐regulation. Together, these hypotheses specify a mediated‐moderation model in which both individual cultural value orientations moderate the relationship between COVID‐19 severity and individual performance through self‐regulation. Therefore:

Hypothesis 4 Through self‐regulation, uncertainty avoidance (H4a), long‐term orientation (H4b), and collectivism (H4c) reduce the negative relationship between COVID‐19 severity and individual performance in GVTs.

## 3 METHOD

### 3.1 Sample and data collection

To test our hypotheses, we used data from an international business consulting project. The participants were undergraduate and graduate students (see Appendix A.1 for a discussion of the use of a student sample in this research context). They worked in GVTs of four to seven people, each team member being from a different country. The GVTs worked on the project for 9 weeks, developing a solution to a real‐life business challenge presented by an existing client company. The dataset was collected from February to April 2020 via weekly online questionnaires and a post‐project questionnaire 2 weeks after the ninth and final week of the project. All participants who had left more than 10% of the questions unanswered were removed from the sample (96% effective response rate, 4% were removed due to incomplete weekly surveys). The final sample comprised 2727 participants working from 31 countries, and covered all 11 cultural clusters identified by Ronen and Shenkar (2013) (see Table A1 for an overview). The sample comprised 54% females. The average age of the participants was 22 years. Almost all participants in the project were in their final year of undergraduate studies or were graduate students. The majority of students were to graduate within a year and would therefore face pandemic‐related challenges and the threat of employee resource loss. Forty‐eight percent of the participants were part‐time or full‐time employed at the time of the survey, and 79% had work experience.

### 3.2 Measures

#### 3.2.1 Cultural value orientation

We used Yoo et al.'s (2011) five‐item measure of uncertainty avoidance (*α* = .78), a six‐item measure of collectivism (*α* = .71), and a six‐item measure of long‐term orientation (*α* = .75) to assess individual cultural value orientations. Sample items for uncertainty avoidance were “It is important to have instructions spelled out in detail so that I always know what I'm expected to do” and “Standardized work procedures are helpful.” Sample items for collectivism were “Individuals should sacrifice self‐interest for the group” and “Individuals should stick with the group even through difficulties.” The response scales for both orientations ranged from 1 (*strongly disagree*) to 5 (*strongly agree*). Sample statements for long‐term orientation were “Personal steadiness and stability,” “Going on resolutely in spite of opposition (Persistence),” “Working hard for success in the future,” and “Long‐term planning.” The response scale for this orientation ranged from 1 (*very unimportant*) to 5 (*very important*). The three orientations were measured during the eighth week of the project.

#### 3.2.2 Self‐regulation

We measured overall self‐regulation (*α* = .84) with 10 items. Specifically, we used Yeo and Frederiks' (2011) instrument to measure the affective and cognitive facets of self‐regulation. Affective self‐regulation (*α* = .77) was measured with three items (e.g., “I try to keep my feelings from interfering too much.”). Cognitive self‐regulation (*α* = .70) was measured with three items (e.g., “I focus my attention on completing a task as best I can.”). Behavioral self‐regulation (*α* = .70) was measured using four items by Brown et al. (1999) (e.g., “I usually judge what I'm doing by the consequences of my actions.”). Self‐regulation was measured during the ninth week of the project with a 5‐point Likert scale from 1 (*strongly disagree*) to 5 (*strongly agree*).

#### 3.2.3 COVID‐19

We used the publicly available COVID‐19 data provided by the Johns Hopkins Coronavirus Resource Center at Johns Hopkins University (Dong et al., 2020) to calculate the relative increase in deaths over a week in each of the 31 countries in our sample. We used COVID‐19 data 5 days before we measured self‐regulation.

#### 3.2.4 Individual performance

Individual performance was assessed based on peer evaluation of the members of a GVT via the weekly surveys conducted during the nine‐week project and via a post‐project survey 2 weeks later (Week 11). In testing the hypotheses, we used the peer evaluations of the post‐project survey. The measure covered five aspects related to both task and contextual performance (e.g., Conway, 1999), specifically effort (“Please evaluate the performance of each of your team members: Was hard‐working.”), quality of ideas (“Please evaluate the performance of each of your team members: Had creative ideas.”), communication (“Please evaluate the performance of each of your team members: Nice, friendly, positive.”), collegiality (“Please evaluate the performance of each of your team members: Worked closely with this person.”), and help with coordinating team efforts (“Please evaluate the performance of each of your team members: Showed leadership, helped with coordination.”). The evaluations were completed on a scale from 1 (poor) to 5 (excellent). The interrater reliability across the peer evaluation performance facets was good and the aggregation of peer evaluations to an individual performance measure was adequate (see Appendix A.2).

#### 3.2.5 Control variables

We included various control variables at the individual and team level, as well as at the country level to rule out alternative explanations (Becker et al., 2016). Previous research has recommended the assessment of cultural value orientations in their entirety (e.g., Hobfoll et al., 2018; Taras et al., 2010). Although people with a high‐power distance may be more likely to self‐regulate when working for leaders who demand high performance (e.g., Matthews et al., 2021), GVTs are often characterized by shared leadership and self‐management (e.g., Hanna et al., 2021; Hertel et al., 2005), and thus, power distance could be less relevant. As a more masculine value orientation is associated with a stronger focus on achievement and performance, people might be more likely to self‐regulate to achieve their individual goals (e.g., Kurman, 2001). We therefore included power distance (five items; *α* = .75) and masculinity (five items; *α* = .70) at the individual level using Yoo et al.'s (2011) measure. The response scales ranged from 1 (*strongly disagree*) to 5 (*strongly agree*). During the eighth week of the project, both cultural value orientations were measured along with the three value orientations used in our hypotheses.

We included participants' age and gender in the analysis because they are related to both self‐regulation and task performance (Kurman, 2001; Ng & Feldman, 2008; Roth et al., 2012). We included participants' level of studies in the analysis as both theoretical arguments and empirical evidence suggest that a higher level of education, and thus more experience with comparable projects, may be associated with higher task performance (e.g., Ng & Feldman, 2008). The official language of the project was English, and all the participants conducted the surveys in English. To account for the potential influence of answering the survey in a foreign language (Harzing, 2005) and for the degree to which language skills were predictive of performance, we assessed English language skills using a 30‐item English proficiency test. In addition, we also asked participants to specify the number of languages that they speak. Theory and empirics indicate that international experience may influence individual performance in GVTs (e.g., Taras et al., 2019). Prior research theoretically argued and empirically showed that work experience and work roles are associated with individual performance (e.g., Ng & Feldman, 2008). Thus, we controlled for work experience and current employment. Previous empirical findings indicate that team size, team time zone differences, and team cultural distance could influence individual team performance in GVTs (e.g., Maynard et al., 2012). Thus, we included these variables in the analysis. Table A2 provides an overview of all control variables assessed based on survey information. Furthermore, we controlled for differences in the way in which the project was included in the course grade and for a potential bias of different universities (e.g., Taras et al., 2021). We also asked participants about the study country, country of origin, and home country. The latter question was used to identify and only include those participants in our sample who selected the same country. All control variables were assessed during the first week of the project.

We included several secondary data control variables related to the COVID‐19 pandemic to rule out potential alternative explanations (see Appendix A.3 for a detailed overview of the measures), among others, a stringency index (three constituting facets, i.e., a containment and health index, an economic support index, and the government response index) and the gross domestic product (GDP) of respondents' country of residence. In addition to the secondary data, we used primary data from the survey to assess three specific aspects related to the pandemic: the perceived general impact of the pandemic on respondents' personal life (general impact), perceived impact on the respondents' jobs (own job impact), and perceived impact on the jobs of respondents' parents (parents' job impact).

## 4 RESULTS

Descriptive statistics, bivariate correlations, and Cronbach's alphas for all variables are presented in Table 1. Table 2 presents the results of the regression analysis.

**TABLE 1:** Descriptive statistics and bivariate correlations

	Variable	*M*	*SD*	1	2	3	4	5	6	7	8	9	10	11	12	13	14	15	16	17	18	19	20	21	22	23	24	25	26	27	28	29	30	31	32	33	34
1.	Age	22.08	4.40																																		
2.	Gender (1 = female)	0.54	0.50	.01																																	
3.	Study level (1 = undergraduate)	0.82	0.38	−.18^***^	.02																																
4.	English proficiency	91.33	11.73	.00	−.02	−.01																															
5.	Languages	1.98	0.98	−.05^**^	.05^**^	−.18^***^	−.07^***^																														
6.	International experience	4.96	1.58	.06^**^	−.01	.02	−.02	.03																													
7.	Work experience	3.30	1.71	.36^***^	−.08^***^	.06^**^	.17^***^	−.31^***^	.01																												
8.	Employment	1.97	1.27	.24^***^	.00	.06^**^	.05^**^	−.19^***^	.05^***^	.54^***^																											
9.	Team size	5.22	0.74	−.02	−.07^***^	.05^**^	.01	−.02	−.01	.03	.00																										
10.	Team cultural distance	1.78	0.02	.01	.03	.04^*^	.01	.03	.03	.02	.02	.05^*^																									
11.	Team time zone difference	5.04	1.61	.03	.02	.01	−.03	.04^*^	.04^*^	.03	.04^*^	.04^*^	.34^***^																								
12.	Percent of course grade	0.33	0.19	.04^*^	.04^*^	−.01	−.08^***^	.06^**^	.05^**^	−.07^**^	−.05^**^	.05^**^	.03	.04^*^																							
13.	Collectivism	3.43	0.68	.02	−.12^***^	−.02	−.02^**^	.03	.08^***^	−.13^***^	−.08^***^	−.01	.01	.05^**^	.10^***^	(.71)																					
14.	Masculinity	2.29	0.88	−.03	−.28^***^	.05^**^	−.06^**^	.01	.10^***^	−.07^**^	−.10^***^	−.05^**^	.05^**^	−.01	.03	.13^***^	(.70)																				
15.	Power distance	1.86	0.72	−.04^*^	−.22^***^	−.03	−.09^***^	−.01	.00	−.02	−.01	−.03	.03	.05^**^	−.04^*^	.00	.46^***^	(.75)																			
16.	Uncertainty avoidance	4.08	0.64	.07^***^	.17^***^	.05^**^	−.01	−.04^*^	.09^***^	−.06^**^	−.03	−.07^**^	.06^**^	.03	.12^***^	.36^***^	−.02	−.24^***^	(.78)																		
17.	Long‐term orientation	4.16	0.57	.06^**^	.07^***^	.02	.02	.01	.09^***^	−.02	.02	−.04^*^	.05^**^	.07^**^	.10^***^	.23^***^	−.08^***^	−.20^***^	.46^***^	(.75)																	
18.	Self‐regulation	3.82	0.59	.05^**^	.04^*^	.00	.04^**^	.00	.07^**^	.01	.00	−.01	.03	.03	.07^**^	.16^***^	−.07^*^	−.20^***^	.34^***^	.38^***^	(.84)																
19.	Affective self‐regulation	3.66	0.75	.06^**^	−.15^***^	.02	.04^*^	−.03	.08^***^	.02	.01	.00	.02	−.01	.06^**^	.14^***^	.03	−.08^***^	.20^***^	.27	.81^***^	(.70)															
20.	Cognitive self‐regulation	4.06	0.73	.03	.07^**^	−.03	.02	.05^**^	.06^**^	−.04^*^	−.02	−.03	.03	.00	.06^**^	.14^***^	−.11	−.23^***^	.34^***^	.38	.87^***^	.55^***^	(.77)														
21.	Behavioral self‐regulation	3.75	0.62	.06^**^	.00	.03	.05^**^	−.01	.05^**^	.05^**^	.02	−.01	.03	−.03	.06^**^	.15^***^	−.03	−.14^***^	.28^***^	.31	.88^***^	.53^***^	.68^***^	(.72)													
22.	COVID‐19 (Δ deaths week in %)	1.26	1.59	−.08^***^	−.01	.11^***^	−.06^**^	.15^***^	.10^***^	−.28^***^	−.07^**^	−.02	.02	−.01	.31^***^	.24^***^	.05^**^	−.08^**^	.23^***^	.21^***^	.13^***^	.12^***^	.13^***^	.08^***^													
23.	COVID‐19 (total deaths log)	3.86	1.18	.06^**^	−.08^***^	−.03	.16^***^	−.37^***^	−.02	.41^***^	.26^***^	.06^**^	.03	−.02	−.21^***^	−.13^***^	−.17^***^	.02	−.14^***^	−.11^***^	−.06^**^	−.04^*^	−.07^**^	−.03	−.37^***^												
24.	COVID‐19 (Δ infections week in %)	0.41	0.28	−.12^***^	.00	.12^***^	−.04^*^	.03	.09^***^	−.18^***^	−.02	−.05^**^	−.03	.03	.14^***^	.08^***^	.07^***^	.00	.13^***^	.08^***^	.05^**^	.04^*^	.05^**^	.03	.37^***^	−.14^***^											
25.	COVID‐19 (total infections log)	4.78	1.20	.07^**^	−.11^***^	−.10^***^	.21^***^	−.42^***^	−.01	.52^***^	.29^***^	.04^*^	.03	−.03	−.20^***^	−.14^***^	−.14^***^	.03	−.15^***^	−.12^***^	−.04^*^	−.02	−.07^**^	−.02	−.42^***^	.90^***^	−.10^***^										
26.	Stringency index	70.19	13.22	−.03	.03	−.30^***^	−.25^***^	.43^***^	−.10^***^	−.36^***^	−.18^***^	−.05^**^	−.03	.02	.03	.07^***^	−.04	.05^**^	−.03	−.01	−.04^*^	−.08^***^	.04^*^	−.05^**^	−.01	−.35^***^	.22^***^	−.41^***^									
27.	Government response index	59.10	12.89	−.04^*^	.07^***^	−.30^***^	−.32^***^	.46^***^	−.08^***^	−.41^***^	−.20^***^	−.05^**^	.02	−.03	.06^**^	.06^**^	−.02	.05^**^	−.03	−.01	−.05^**^	−.09^***^	.04^*^	−.06^**^	.07^***^	−.41^***^	.14^***^	−.51^***^	.94^***^								
28.	Containment and health index	62.72	11.11	−.02	.06^**^	−.33^***^	−.28^***^	.39^***^	−.09^***^	−.34^***^	−.15^***^	−.04^*^	−.01	−.02	.02	.05^**^	−.07^***^	.05^**^	−.04^*^	−.01	−.05^**^	−.10^***^	.03	−.06^**^	−.03	−.21^***^	.18^***^	−.32^***^	.94^***^	.96^***^							
29.	Economic support index	33.73	35.38	−.07^***^	.07^***^	−.14^***^	−.30^***^	.49^***^	−.03	−.45^***^	−.24^***^	−.07^**^	−.03	.03	.13^***^	.08^***^	.09^***^	.03	.00	−.01	−.03	−.06^**^	.03	−.04^*^	.28^***^	−.74^***^	.00	−.78^***^	.67^***^	.80^***^	.61^***^						
30.	GDP (log)	6.37	1.02	.04^*^	−.09^***^	−.04^*^	.19^***^	−.45^***^	.00	.49^***^	.27^***^	.05^**^	.02	−.01	−.23^***^	−.16^***^	−.13^***^	.04^*^	−.15^***^	−.13^***^	−.05^**^	−.03	−.09^***^	−.03	−.44^***^	.90^***^	−.10^***^	.96^***^	−.49^***^	−.56^***^	−.38^***^	−.80^***^					
31.	COVID‐19 impact on general life	4.62	2.25	−.02	.10^***^	−.03	.08^***^	−.06^**^	−.05^**^	.03	.02	.00	.03	.01	.01	.04	−.12^***^	−.12	.12^***^	.10^***^	.10^***^	.05^**^	.11^***^	.09^***^	−.02	.02	.01	.02	.03	.00	.03	−.05^**^	−.01				
32.	COVID‐19 impact on own job	2.87	2.36	.04^*^	.02	.03	−.01	−.12^***^	.02	.25^***^	.33^***^	−.00	.01	.00	−.06^**^	−.05^**^	−.03	.00	−.02	−.01	−.03	−.03	−.03	−.02	−.11^***^	.17^***^	.00	.17^***^	−.06^**^	−.06^**^	−.04^*^	−.11^***^	.18^***^	.05^**^			
33.	COVID‐19 impact on parents' job	3.14	2.09	−.17^***^	.04^*^	.03	−.06^**^	.04^*^	.03	−.13^***^	−.09^***^	−.02	.02	−.00	.08^***^	.10^***^	.01	−.02	.08^***^	.09^***^	.04	.02	.06^**^	.03	.14^***^	−.11^***^	.08^***^	−.12^***^	.07^***^	.09^***^	.07^***^	.10^***^	−.12^***^	.12^***^	.14^***^		
34.	Individual performance	4.14	0.65	.01	.12^**^	−.05^**^	.12^**^	.02	−.07^**^	.08^***^	.00	−.02	−.04^*^	−.02	−.03	−.04^*^	−.17^**^	−.15^**^	−.01	.06^**^	.08^***^	.02	.09^***^	.08^***^	−.14^***^	.09^***^	−.11^***^	.11^***^	.02	.00	.03	−.07^***^	.09^***^	.08^***^	−.02	−.05^***^	(.91)

*Note*: *N* = 2727. The figures in parentheses are Cronbach's alphas. Gender: 1 = female, 0 = male. Study level: 1 = undergraduate, 0 = graduate.

* *p* < .05.

** *p* < .01.

*** *p* < .001.

**TABLE 2:** Regression results for testing mediation and moderation

Variable and statistics	Individual performance												Self‐regulation					
	Model 1			Model 2			Model 3			Model 4			Model 5			Model 6		
	*B*	(*p*)	*SE*	*B*	(*p*)	*SE*	*B*	(*p*)	*SE*	*B*	(*p*)	*SE*	*B*	(*p*)	*SE*	*B*	(*p*)	*SE*
Controls																		
Age	−.01	(.545)	.00	−.01	(.458)	.00	−.01	(.403)	.00	−.01	(.403)	.00	.01	(.000)***	.00	.01	(.058)	.00
Gender (1 = female)	.12	(.000)***	.03	.14	(.000)***	.03	.13	(.000)***	.03	.13	(.000)***	.03	−.09	(.000)***	.02	−.11	(.000)***	.01
Study level (1 = undergraduate)	−.01	(.769)	.06	−.01	(.804)	.06	−.01	(.817)	.06	−.01	(.861)	.06	−.01	(.957)	.05	−.01	(.813)	.04
English proficiency	.01	(.001)**	.00	.01	(.001)**	.00	.01	(.001)**	.00	.01	(.001)**	.00	.00	(.072)	.00	.00	(.159)	.00
Languages	.03	(.051)	.02	.03	(.063)	.02	.03	(.045)*	.02	.03	(.048)*	.02	.03	(.953)	.01	.02	(.080)	.01
International experience	−.02	(.008)**	.01	−.02	(.002)**	.01	−.02	(.008)**	.01	−.02	(.006)**	.01	.02	(.023)*	.01	.02	(.157)	.01
Work experience	.04	(.067)	.02	.04	(.066)	.02	.04	(.076)	.02	.04	(.077)	.02	.02	(.715)	.01	.01	(.192)	.01
Employment	−.03	(.004)**	.01	−.03	(.002)**	.01	−.03	(.009)**	.01	−.03	(.013)*	.01	.01	(.789)	.01	−.01	(.465)	.01
Team size	−.02	(.154)	.02	−.02	(.172)	.02	−.02	(.173)	.02	−.02	(.182)	.02	−.01	(.277)	.01	.01	(.819)	.01
Team cultural distance	−.01	(.351)	.00	−.02	(.382)	.00	−.02	(.383)	.00	−.02	(.391)	.00	.01	(.153)	.00	.01	(.230)	.00
Team time zone difference	−.01	(.154)	.00	−.02	(.172)	.00	−.02	(.173)	.00	−.02	(.182)	.00	.01	(.277)	.00	.01	(.819)	.00
Percent of course grade	.02	(.848)	.09	.01	(.943)	.09	.01	(.527)	.09	.01	(.560)	.09	.14	(.025)*	.06	.05	(.410)	.06
Masculinity	−.05	(.042)*	.02	−.05	(.045)*	.02	−.05	(.038)*	.02	−.05	(.036)*	.02	.01	(.427)	.01	−.01	(.930)	.01
Power distance	−.08	(.002)**	.02	−.08	(.007)**	.02	−.08	(.005)**	.02	−.08	(.005)**	.02	−.15	(.000)***	.02	−.07	(.000)***	.02
Stringency index	.01	(.026)*	.00	.01	(.031)*	.00	.01	(.063)	.00	.01	(.050)	.00	−.01	(.452)	.00	−.01	(.379)	.00
GDP	.08	(.009)**	.03	.08	(.007)**	.03	.04	(.145)	.03	.08	(.115)	.03	−.05	(.304)	.05	−.01	(.597)	.02
COVID‐19 impact on general life	.01	(.130)	.01	.01	(.161)	.01	.01	(.129)	.01	.01	(.146)	.01	.02	(.001)**	.01	.01	(.031)*	.01
COVID‐19 impact on own job	−.01	(.223)	.01	−.01	(.225)	.01	−.01	(.221)	.01	−.01	(.229)	.01	−.01	(.172)	.01	−.01	(.213)	.00
COVID‐19 impact on parents' job	−.01	(.002)**	.00	−.01	(.002)**	.00	−.01	(.003)**	.00	−.01	(.002)**	.00	.01	(.170)	.01	.00	(.653)	.00
Direct effects																		
Uncertainty avoidance				−.07	(.000)***	.02	−.06	(.000)***	.02	−.06	(.000)***	.02				.16	(.000)***	.01
Long‐term orientation				.07	(.001)**	.02	.08	(.000)***	.02	.08	(.000)***	.02				.28	(.000)***	.02
Collectivism				.02	(.356)	.02	.02	(.168)	.02	.02	(.219)	.02				.03	(.143)	.02
Self‐regulation				.07	(.013)*	.03	.07	(.013)*	.03	.04	(.000)***	.03						
COVID‐19 (Δ deaths week in %)							−.04	(.024)*	.02	−.07	(.000)*	.02						
Moderation effect																		
Self‐regulation × COVID‐19 (Δ deaths week in %)										.03	(.000)***	.01						
Intercept	3.14	(.000)***	.34	2.80	(.000)***	.33	3.13	(.000)***	.31	3.30	(.000)***	.32	4.06	(.000)***	.54	1.96	(.000)***	.23
*F*		71.99	(.000)***		152.07	(.000)***		164.96	(.000)***		142.68	(.000)***		77.37	(.000)***		308.05	(.000)***
*R* ^2^		.078			.087			.092			.094			.063			.19	
Adjusted *R* ^2^		.072			.079			.084			.086			.057			.19	
Change in *R* ^2^					.009			.005			.002						.14	

*Note*: *N* = 2727. Unstandardized estimates. Cluster‐robust standard errors for country (*N* = 31). Gender: 1 = female, 0 = male. Study level: 1 = undergraduate, 0 = graduate. Exact *p* values in parentheses.

* *p* < .05.

** *p* < .01.

*** *p* < .001.

While our hypotheses were developed at the individual level of analysis, COVID‐19 data are reported at the country level. Therefore, we used cluster‐robust standard errors for countries (McNeish et al., 2017). Models 1 to 4 present the results for individual performance, and Models 5 and 6 present the results for self‐regulation. Model 1 shows that nine of the 19 control variables are significantly associated with performance. While female respondents (*B* = .12, *p* = .000) and respondents with higher English proficiency levels (*B* = .01, *p* = .001) achieved higher performance, respondents with higher international experience (*B* = −.02, *p* = .008), masculinity values (*B* = −.05, *p* = .042), and higher power distance values (*B* = −.08, *p* = .002) achieved lower individual performance. Respondents who reported a stronger impact of the pandemic on their parents' or guardians' jobs achieved lower performance (*B* = −.01, *p* = .002). At the country level, a higher stringency index (*B* = .01, *p* = .026) and a higher GDP (*B* = .08, *p* = .009) resulted in higher performance. Together, the control variables accounted for 8% of the variance in individual performance. We included the three cultural value orientations and self‐regulation in Model 2. While a higher uncertainty avoidance resulted in lower performance (*B* = −.07, *p* = .000), a higher long‐term orientation resulted in higher performance (*B* = .07, *p* = .001). Respondents who reported higher self‐regulation achieved a higher performance (*B* = .07, *p* = .013).

Hypothesis 1 states that COVID‐19 severity is negatively associated with individual performance. In support of Hypothesis 1, Model 3 shows that the relationship between COVID‐19 severity and individual performance is negative and significant (*B* = −.04, *p* = .024). In the weekly survey, all participants also gave qualitative feedback on the progress of the project (“In your own words, please describe how the project has been going on for you so far.”). The influence of the pandemic on the performance of the team members was also evident in these comments, with pandemic‐related stress, problems, and difficulties being the top three categories overall. Table 3 presents content analysis results for comments that referred to COVID‐19. The ambient stress—created by the pandemic—was apparent in a large number of comments. For example, “At first, we were all very happy and excited to work on the project but then each one of us faced a problem ‐ COVID‐19. I mean, it was and still is the cause of all problems. It is very difficult to organize your time when everyone is stressing out.” Another participant mentioned: “In addition, the situation with COVID‐19 made the cooperation more difficult in the sense that maybe everyone suffered from a certain stress and the motivation was not high due to the initial restrictions.” Another participant stated: “The quality of work between all team member is not the same. We can still deliver a good report but it is obvious that this is not a priority for anybody, mostly because of the already high stress levels caused by the pandemic.” A participant elaborated on this: “The stressors of the current situation of the national pandemic and the fact that my household, like many others, have been directly affected has made schoolwork a bit more difficult. Staying on task has been extremely difficult as so many other things are going on.” A participant wrote: “I have had a stressful week dealing with the coronavirus and have not been able to apply a lot of time to the project,” as did two other participants in a similar vein: “It has been a very stressful week. People are dying, and my family is in danger” and “I am really stressed with the whole world situation so my focus is off. I am worried about my daughter and her possibly being sick so working on the project hasn't been my first priority.” In sum, both the quantitative and the qualitative results illustrate how the pandemic negatively influences individual performance.

**TABLE 3:** Summary of pandemic‐related content in respondents' weekly project comments

Pandemic‐related search keywords							Results of content analysis of pandemic‐related comments							
Week	Comments in total	Pandemic‐related comments	Comments referring to “COVID‐19”/“COVID”	Comments referring to “Corona”/“virus”	Comments referring to “Pandemic”	Comments referring to “World” or “Situation” in the pandemic context	Stress	Anxiety/scared/fear	Problems	Difficult time	Impact on studies	Sick/infected	Worried/concerns	Information exchange
2	1427	2 (0.2)	0	2	0	0	0	0	0	0	2	0	0	0
3	1987	24 (1.2)	4	21	0	0	0	1	0	2	7	0	7	11
4	2019	151 (7.5)	18	98	6	25	9	4	26	61	38	0	9	11
5	2143	258 (12.0)	46	134	30	48	32	11	53	27	37	5	20	37
6	2097	156 (7.4)	48	48	23	29	22	6	38	14	21	8	11	12
7	2014	81 (4.0)	38	16	14	13	15	3	19	17	14	5	0	0
8	1923	70 (3.6)	20	18	21	11	11	2	21	18	7	5	0	0
11	2160	465 (21.5)	214	73	107	71	79	9	134	117	46	12	7	0
Total	15,770	1207 (7.7)	398	394	210	195	170	38	294	204	159	39	60	92

*Note*: Total number of words: 425,485. Numbers in parentheses are the percentage of the pandemic‐related comments of the comments in total.

**FIGURE 2:**
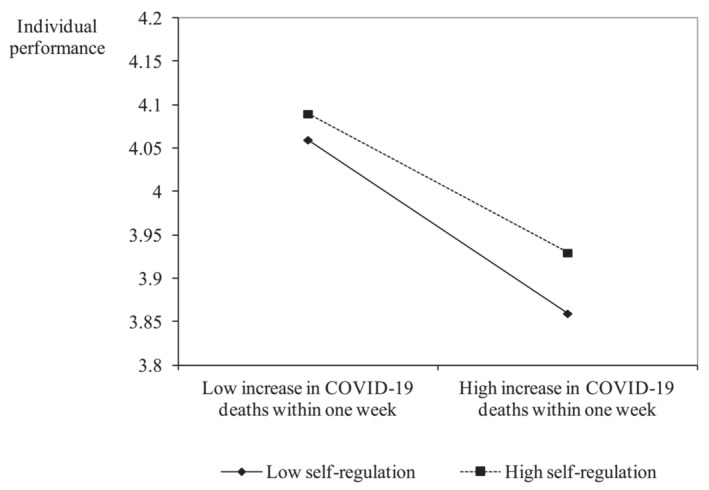
Moderation effect between COVID‐19 and self‐regulation on individual performance

Hypothesis 2 states that the negative relationship between COVID‐19 severity and individual performance is weaker for individuals high in self‐regulation than for those low in self‐regulation. Model 4 shows that self‐regulation moderates the relationship between COVID‐19 severity and individual performance in the hypothesized way (*B* = .03, *p* = .000). Figure 2 (Dawson, 2014) shows that while the relationship between COVID‐19 severity and individual performance is negative for both low and high levels of self‐regulation, self‐regulation reduces the negative effect of COVID‐19 severity.

Although none of the participants specifically referred to self‐regulation, the empirical results are reflected in participants' comments, indirectly referring to coping and self‐control, allowing them to move on despite the pandemic. A participant wrote: “I have also learnt to cope with difficult situations since I encountered some difficulties along the line.” Another participant mentioned: “I found out that I have great self‐control,” and yet another stated that: “The project is becoming tougher but I'm trying to cope.” A participant reported: “It has been very tough […] this deadly virus has made our project difficult but we are coping with the situation.” Another participant was more positive: “I believe that my teammates and I will cope with everything,” as was the participant who observed: “Even during this rough phase of pandemic, we are able to cope up well and do a good job.” In a similar one participant wrote: “Dealing with COVID‐19 is stressful, so it makes other activities more stressful. I am coping as best that I can.” Another participant emphasized: “Yes, there are some problems for each of us, but we will cope with everything! The main thing is to set a goal and strive for it!” In sum, both the quantitative and qualitative data support our argument.

Models 5 and 6 report the results of self‐regulation. Model 5 shows the results of the control variables. The results showed a positive association between age (*B* = .01, *p* = .000), international experience (*B* = .02, *p* = .023), and percentage of course grade and self‐regulation (*B* = .14, *p* = .025). Furthermore, the results confirm a negative relationship between gender and self‐regulation (*B* = −.09, *p* = .000), as well as between power distance and self‐regulation (*B* = −.15, *p* = .000). Hypothesis 3 states that uncertainty avoidance (H3a), long‐term orientation (H3b), and collectivism (H3c) are positively associated with self‐regulation. In support of Hypotheses 3a and 3b, Model 6 shows that both uncertainty avoidance (*B* = .16, *p* = .000) and long‐term orientation (*B* = .28, *p* = .000) are positively and significantly associated with self‐regulation. The results show that collectivism is not significantly related to self‐regulation (*B* = .03, *p* = .143), rejecting Hypothesis 3c.

Hypothesis 4 posits that uncertainty avoidance (H4a), long‐term orientation (H4b), and collectivism (H4c) reduces the negative relationship between COVID‐19 and individual performance through self‐regulation. Table 4 shows the results of the additional analysis of the indirect moderating effect of the three cultural value orientations through self‐regulation. The three indirect moderation effects are positive and significant in support of Hypotheses 4a, 4b, and 4c.

**TABLE 4:** Conditional effects of uncertainty avoidance, long‐term orientation, and collectivism through self‐regulation

Variable	Coefficient	*SE*	*z*	*p*	95% CI
Uncertainty avoidance					
*M* − 1*SD*	.014	.004	3.19	.001	.005/.022
*M*	.022	.007	3.42	.001	.009/.035
*M* + 1*SD*	.031	.010	2.97	.003	.010/.051
Long‐term orientation					
*M* − 1*SD*	.021	.007	3.16	.002	.008/.034
*M*	.034	.010	3.39	.001	.014/.054
*M* + 1*SD*	.047	.016	2.94	.003	.016/.078
Collectivism					
*M* − 1*SD*	.013	.005	2.86	.004	.006/.024
*M*	.015	.004	4.12	.000	.009/.024
*M* + 1*SD*	.016	.004	4.01	.000	.009/.025

*Note*: *N* = 2727. The results were based on 10,000 bootstraps. Cluster‐robust standard errors clustered for country (*n* = 31). Bias‐corrected confidence intervals (CI).

Although the GVT members' open text comments were not directly related to cultural values as a determinant of self‐regulation, the members recognized that individuals in different countries handled the situation differently, for example, “[…] everyone is unfortunately dealing with this crisis differently.” Another participant wrote “I think within my group there has been a bit of confusion because of the coronavirus affecting us differently.” A participant elaborated on this: “We are all trying to get things together, regardless of the Coronavirus that has impacted everyone differently (might cause stress, fear, worries, etc.).” Another participant explained: “This past week has been difficult because of the effects of the Corona virus. Three of my teammates have been struggling personally in different ways, while my other US teammate and I have worked to try to maintain some order and direction. […] I have been able to cope with this, it seems, slightly better than some of my teammates.”

We conducted several robustness checks to assess the sensitivity of our results. First, removing the control variables (e.g., Becker et al., 2016) did not affect the statistical significance of our findings, and the reported main coefficient was comparable in effect size (see Table A3). Second, instead of the increase in COVID‐19‐related deaths within 1 week, we also examined the logarithmized absolute number of COVID‐19‐related deaths, the logarithmized absolute number of COVID‐19 cases, and the increase of COVID‐19 infections within 1 week. While the increase in COVID‐19 infections within 1 week showed a significant negative effect on performance, the total number of COVID‐19‐related deaths and infections had no significant effect on performance. The results for the direct effect of the COVID‐19 pandemic and the moderating role of self‐regulation did not remain significant for these alternative measures. The number of people who had tested positive for and the number of people who had died from COVID‐19 were most often reported data in mass media and public health communications at the onset of the pandemic. Our results show that, specifically, the increase in COVID‐19 related deaths seemed to be in individuals' attentional focus and that this affected individual performance. A potential explanation for this finding is that specifically during the early phase of the pandemic and due to the limited knowledge and understanding of COVID‐19, the uncertainty related to the threat of a dying from it was high. As a result, individuals focused on the number of deaths to be able to evaluate the severity of this disease. Third, we examined the results for each of the three self‐regulation facets individually. While the results for emotional self‐regulation did not support our hypotheses, the results for cognitive and behavioral self‐regulation were in line with the findings for overall self‐regulation. In the open text comments participants referred specifically to the challenges and difficulties created by the pandemic and less often to emotional reactions, such as anxiety, fear, panic, and worrying. While previous studies highlighted the importance of emotion regulation in GVTs (e.g., Ayoko et al., 2012), our results suggest that at the onset of the pandemic and in the specific context of the consulting project, self‐regulation of emotions was not effective in buffering the adverse effects of the pandemic.

## 5 DISCUSSION

The persistent ambient stress caused by the COVID‐19 pandemic has had an enormous impact on individuals and their ability to fulfill their tasks and responsibilities. In this study, we developed and examined a conceptual model in which the local severity of the pandemic has had negative effects on the performance of individuals in GVTs. This effect is reduced by self‐regulation, which in turn is driven by a set of cultural value orientations. The results of a longitudinal study combining individual‐level and country‐level data for a sample of 2727 individuals located in 31 countries, and working on an international consulting project in GVTs, show that self‐regulation acts as a buffer against the adverse effects of the pandemic on peer‐assessed individual performance. Our findings contribute to a better theoretical understanding as they inform the individual‐level performance consequences of a pandemic. Along with the identification of an effective coping strategy, they help to mitigate long‐term, negative work‐related implications. Furthermore, our findings illustrate the cultural embeddedness of self‐regulation and cross‐cultural differences in individuals' self‐regulation.

### 5.1 Implications for theory

Our results contribute to the literature in three ways. First, by building on COR theory and answering scholarly calls for empirical investigations of the pandemic‐related antecedents of individual performance in (global virtual) teams (Caligiuri et al., 2020; Collings et al., 2021; Kniffin et al., 2021), we advocate that the COVID‐19 pandemic negatively influences individuals' performance in teams, specifically in GVTs. The existing literature on major technological and natural disasters and their influence on individual performance mainly focuses on the performance of individuals working in the healthcare sector. We still have a limited understanding of the influence of such highly disruptive events on individuals' performance in other contexts. In line with COR theory, our results show that the pandemic, when accessed via the relative increase of COVID‐19‐related deaths within a week, has a negative effect on the performance of people working in a team context, especially in GVTs. The results of our study are in line with recent findings on the negative influence of the pandemic on individual performance in general (Yi‐Feng Chen et al., 2021) and further relate to recent research that has shown that specific remote work challenges are negatively related to individual performance during the pandemic (Wang et al., 2020). We extend this important research by focusing on a specific form of individual performance, namely, the performance of individuals working in GVTs. Scholars have highlighted the importance of GVTs during the pandemic (Caligiuri et al., 2020; Klonek et al., 2021; Kniffin et al., 2021). It is important to distinguish between GVTs and traditional co‐located team structures because these teams differ substantially in how joint goals are achieved (Jimenez et al., 2017; Martins et al., 2004).

Second, integrating the main tenets of COR theory and self‐regulation theory, we theoretically identified and empirically showed that individuals' self‐regulation functions as a moderator of the adverse relationship between the COVID‐19 pandemic and individual performance in teams, specifically in GVTs. Combining both theories into a cohesive understanding of the role of self‐regulation, our results answer recent calls to identify specific coping strategies in a performance context during major disasters, such as the COVID‐19 pandemic (Hobfoll et al., 2018). We highlight the buffering effect of self‐regulation and the link to emerging research on self‐regulation in organizations (Burnette et al., 2013; Wood, 2005). Specifically, our study extends related work (Niessen & Jimmieson, 2016; Porath & Bateman, 2006) as called for by several scholars (e.g., Lian et al., 2017), by showing that self‐regulation also matters for performance in the context of teams, specifically in GVTs. Thus, our study contributes to theory development on the effects of self‐regulation in the workplace, which has suffered from limited theoretical grounding and empirical testing (Boekaerts et al., 2005; Kanfer, 2005; Vancouver & Day, 2005).

Third, drawing from theories of cultural values and a cultural‐embeddedness perspective of self‐regulation, we link individuals' cultural value orientations to self‐regulatory processes, thereby providing theoretical insights into and empirical findings on the cultural origins of coping strategies such as self‐regulation (Hobfoll, 2002; Hobfoll et al., 2018; Salas et al., 2017). Hobfoll (1988, 2001, 2012) emphasized the importance of considering coping strategies within their cultural context. Previous research noted that although self‐regulation plays a key role in fulfilling tasks and obligations, and therefore, in achieving goals, few culture‐informed studies exist on the developmental drivers of self‐regulation related to achievement (Trommsdorff & Cole, 2011). Therefore, it is theoretically important to explain how individuals' cultural backgrounds influence self‐regulation. Our study reveals that both uncertainty avoidance and long‐term orientation are positively associated with self‐regulation and that self‐regulation partially mediates the associations between individual cultural value orientations and individual performance. Our research brings together two key aspects of COR theory: (1) Individuals need to invest resources to protect the resources they value, considering that coping strategies, such as self‐regulation, enable individuals to protect their resources. (2) Self‐regulation is embedded in a cultural framework, contributing to a more complete understanding of how such resources are culturally embedded. Accordingly, theorizing on self‐regulation should be characterized by acknowledging that individuals' efforts to modify affective and cognitive processes as well as behaviors to achieve goals are immersed in cultural values.

In contrast to our hypotheses, we found no significant influence of collectivism on self‐regulation. Furthermore, our results revealed a negative relationship between power distance and self‐regulation. At the individual level, power distance refers to “the extent to which an individual accepts the unequal distribution of power in institutions and organizations” (Clugston et al., 2000, p. 9). Individuals socialized in a cultural context with a high‐power distance expect to be told what to do instead of proactively adapting to challenges. Furthermore, individuals with a high‐power distance orientation are more likely to adopt a (team) hierarchy where everybody has a place and where responsibilities are centralized. Thus, an explanation for our finding could be that individuals high in power distance are likely to be less reliant on the self‐regulation of their performance, as they feel less responsible and are compelled to protect and secure the interests of the group.

### 5.2 Implications for practice

The results of our study do offer practical implications for individuals and organizations facing major external crises, such as the COVID‐19 pandemic. Self‐regulation can be developed through a variety of techniques, and organizations can help individuals build, use, continually improve, and maintain self‐regulation and, in turn, foster resilience to pandemic‐related stress. Organizations can also prepare themselves for pandemic situations by supporting the development of individual resilience, leading to stronger organizational resilience (Kuntz et al., 2016). Specific interventions tailored for GVT members may create an awareness of specific self‐regulation strategies and when to apply these strategies in the work environment, considering the specific work conditions of a pandemic. Individuals vary noticeably in their reactions to pandemic‐related threats (Taylor, 2019), and on the needs to consider when designing interventions to promote self‐regulation. The results of this study show that the individual cultural values of uncertainty avoidance and long‐term orientation are positively related to performance via self‐regulation, while the relationship is negative for power distance. This implies that individuals with a high‐power distance orientation may require more support during a crisis. McEwen (2011) proposes that resilience‐building should focus on five elements: mental toughness, physical balance, emotional endurance, purpose, and meaning. Most of these elements can be addressed through well‐being initiatives in the workplace (Stokes et al., 2019), and they should be part of training and development programs (Ollier‐Malaterre, 2010).

### 5.3 Limitations and future research directions

The findings of this study are subject to several limitations. First, we did not directly assess the pandemic‐related ambient stress of GVT members. While recent research indicates that the proxy we use for pandemic‐related stress in individuals (i.e., change in COVID‐19‐related deaths over a week) is positively related to anxiety and stress during the pandemic (e.g., Hu et al., 2020), future studies should examine the dynamic relations between pandemic‐related ambient stress, self‐regulation, and individual performance over time. The specific scales that have been developed to assess COVID‐19‐related stress provide a fertile basis for future research (e.g., Taylor et al., 2020). Second, while our results support the hypothesized relationships, the effect of self‐regulation is relatively small, and self‐regulation was not able to fully buffer the negative effect of pandemic severity in this GVT context. Thus, the results of this study should be interpreted in light of this, specifically given the relatively large sample size (e.g., Paterson et al., 2016). Furthermore, individual performance in general, and specifically in the context of GVT and a pandemic, is an outcome of complex interactions of various factors, often resulting in relatively small effect sizes (e.g., Götz et al., 2022). In the present study, self‐regulation accounted for additional variance in individual performance over and above this comprehensive set of established predictors of individual performance in GVT. This effect was relatively small on average for an individual during the specific time point at the onset of the pandemic. But, given the duration of the pademic and the large number of individuals working from home around the world, it most likely accumulated to a considerable degree over this longer period of time (e.g., Cortina & Landis, 2009). Third, although self‐regulation has several facets (e.g., Bagozzi, 1992), this study focused only on three facets (affective, cognitive, and behavioral self‐regulation). Future studies could examine whether the direct, moderating, and mediating effects of self‐regulation vary with other self‐regulation facets, such as social and perceptual self‐regulation. Fourth, as the present study's participants were students who worked voluntarily in GVTs, caution is expressed when generalizing findings to corporate employees who must work virtually because of employer recommendations or government regulations. Fifth, we did not ask participants about their marital status or children and how COVID‐19 affected these close contacts. These factors may increase the stress levels of those working in GVTs. Furthermore, we investigated the members of GVTs who participated in a consulting project; a project providing the context for all GVTs. Hence, certain characteristics of the project context may have had an impact on the results. Although we are confident that our findings have theoretical and practical implications for different contexts, replications in other settings are warranted. Sixth, pandemics are dynamic events. Our study provides a snapshot of the time at the onset of the pandemic and does not account for the role of coping strategies at different stages of the pandemic and for the development of these strategies over time (e.g., Bolino et al., 2012). Therefore, we encourage future research on dynamic processes related to self‐regulation in a performance context (Vancouver, 2008). Finally, self‐regulatory abilities may have a promotion or prevention focus. In this study, the outcome variable was individuals' performance in a consulting project. We examined the role of promotion‐focused self‐regulatory abilities. Future studies should compare the distinct functions of promotion‐focused and prevention‐focused self‐regulatory abilities. Despite the limitations of this study, our findings embody an important albeit initial theoretical and empirical step to better understand whether and to what extent the COVID‐19 pandemic influences individuals' performance in GVTs and how culturally embedded self‐regulation abilities enable individuals to cope with these adverse effects.

## CONFLICT OF INTEREST

No conflict of interest.

## Data Availability

The data that support the findings of this study are available from the corresponding author upon reasonable request.

## Footnotes

^1^
We focus on this set of cultural value orientations and include power distance orientation and masculinity/femininity as control variables in the test of the hypotheses as the cultural values underlying power distance and masculinity/femininity should be less relevant in the formation of self‐regulation. At the societal level, power distance refers to the extent to which a society accepts that power is unequally distributed in institutions and organizations (Hofstede, 2001). At the individual level, power distance refers to the extent to which an individual accepts such inequalities (Farh et al., 2007). Thus, individuals with a high‐power distance orientation are likely to accord more value to status, power, and prestige—aspects that are not specifically involved in the formation of self‐regulation coping strategies during the socialization process of individuals. At the societal level, masculinity versus femininity refers to the extent to which a society values more “masculine” traits such as assertiveness and ambition or more “feminine” traits such as an emphasis on interpersonal relationships, helping others, and quality of life (Hofstede, 2001). At the individual level, a higher masculinity value orientation means that challenge and recognition are important and that the emphasis is on advancement, equity, and performance (Hofstede, 2001). These aspects should be less salient in the formation of self‐regulation coping strategies during individuals' socialization process.

## APPENDIX A.

### A..1

**TABLE A1:** Summary of sample characteristics across countries

Country	Sample size	Females (percent)	Mean age (*SD*)	Under‐graduates (percent)	Mean self‐regulation (*SD*)	Cultural cluster	COVID‐19 infections	COVID‐19 deaths	COVID‐19 recovered	Stringency index	Containment and health index	Economic support index	Government response index
Brazil	84	39 (46)	20.86 (2.89)	84 (100)	3.88 (0.55)	Latin America	43,079	2769	22,991	74.54	66.07	50.00	64.06
Canada	12	5 (42)	23.83 (2.33)	0 (0)	3.52 (0.69)	Anglo	39,401	1892	13,188	72.69	61.90	62.50	61.98
China	23	20 (87)	20.09 (1.86)	23 (100)	3.54 (0.58)	Confucian	83,853	4512	77,799	56.94	59.23	12.50	53.39
Colombia	174	118 (68)	21.49 (3.18)	171 (98)	3.83 (0.61)	Latin America	4149	196	804	84.26	80.36	75.00	79.69
Croatia	24	17 (71)	21.75 (1.66)	24 (100)	3.68 (0.64)	East Europe	1908	48	801	96.30	80.95	87.50	81.77
Czech Republic	15	8 (53)	21.20 (5.92)	0 (0)	3.67 (0.66)	East Europe	7033	204	1753	60.19	63.10	100.00	67.71
Ecuador	2	1 (50)	19.00 (1.41)	0 (0)	3.43 (0.20)	Latin America	10,398	520	1207	93.52	78.57	75.00	78.12
Ghana	238	124 (52)	21.09 (2.75)	238 (100)	4.20 (0.53)	African	1042	11	99	62.04	54.17	50.00	53.65
Grenada	41	35 (85)	25.07 (7.74)	41 (100)	4.10 (0.48)	‐	14	0	6	‐	‐	‐	‐
India	74	44 (59)	21.82 (1.31)	6 (8)	4.04 (0.51)	Far East	20,080	645	3975	96.30	89.58	75.00	87.76
Italy	135	68 (50)	23.39 (3.56)	16 (12)	3.88 (0.56)	Latin Europe	183,957	24,648	51,600	93.52	85.42	50.00	80.99
Jamaica	6	4 (67)	22.67 (2.66)	6 (100)	3.69 (0.53)	Far East	223	7	27	80.56	70.24	50.00	67.71
Latvia	10	6 (60)	23.80 (4.85)	10 (100)	3.90 (0.53)	East Europe	748	13	133	69.44	57.74	87.50	61.46
Lithuania	46	23 (50)	22.13 (3.06)	46 (100)	3.62 (0.54)	East Europe	1350	38	298	81.48	66.67	75.00	67.71
Mexico	70	40 (57)	20.83 (2.03)	70 (100)	4.00 (0.54)	Latin America	8772	857	2627	82.41	67.56	0.00	59.11
Netherlands	84	27 (32)	20.25 (1.49)	84 (100)	3.82 (0.39)	Nordic	34,134	4054	250	78.70	63.69	87.50	66.67
Norway	21	9 (43)	24.52 (2.36)	0 (0)	3.85 (0.78)	Nordic	7191	182	32	71.30	51.79	37.50	50.00
Oman	28	12 (43)	21.93 (0.86)	28 (100)	3.90 (0.74)	Arab	1508	10	238	92.59	79.17	62.50	77.08
Pakistan	50	17 (34)	20.80 (3.26)	50 (100)	3.88 (0.70)	Far East	9565	209	2073	92.59	67.86	50.00	65.62
Poland	11	7 (64)	26.09 (3.45)	0 (0)	3.66 (0.52)	East Europe	9856	404	1297	83.33	67.26	37.50	63.54
Portugal	59	41 (69)	23.44 (6.55)	0 (0)	4.01 (0.46)	Latin Europe	21,379	785	917	82.41	70.83	75.00	71.35
Romania	1	1 (100)	23.00 (−)	0 (0)	4.57 (na)	East Europe	9242	508	2153	87.04	69.05	87.50	71.35
Russia	85	63 (74)	20.27 (2.01)	78 (92)	3.69 (0.63)	East Europe	52,763	513	3873	85.19	79.46	50.00	75.78
Saudi Arabia	2	2 (100)	20.50 (0.71)	2 (100)	3.14 (0.00)	Arab	11,631	109	1640	91.67	75.60	62.50	73.96
Switzerland	20	14 (72)	25.04 (4.24)	20 (100)	3.98 (0.59)	Germanic	28,063	1478	19,400	73.15	60.12	62.50	60.42
Taiwan	33	27 (82)	20.70 (1.19)	33 (100)	3.62 (0.59)	Confucian	426	6	307	31.48	33.93	37.50	34.38
Thailand	131	89 (68)	21.47 (2.31)	131 (100)	3.78 (0.62)	Far East	2826	49	2352	76.85	66.96	100.00	71.09
Turkey	14	4 (29)	21.21 (1.48)	14 (100)	3.95 (0.48)	Near East	95,591	2259	14,918	75.93	65.48	62.50	65.10
United Arab Emirates	10	4 (40)	19.60 (2.11)	10 (100)	3.97 (0.53)	Arab	7755	46	1443	87.04	79.76	50.00	76.04
United Kingdom	18	11 (61)	23.56 (1.58)	0 (0)	3.78 (0.64)	Anglo	130,184	17,337	640	79.63	60.71	100.00	65.62
United States of America	1239	607 (49)	22.50 (5.30)	1090 (88)	3.86 (0.64)	Anglo	825,306	56,634	75,673	72.69	66.67	62.50	66.15

*Note*: Cultural clusters are based on Ronen and Shenkar (2013). All COVID‐related data are given for the fifth week of the data collection.

**TABLE A2:** Control variables included in the survey and their measures

Control variable	Measure
Age	Age was measured as a continuous variable in years
Gender	Gender was measured as a dichotomous variable coded “1” for females and “0” for males
Study level	Level of studies was measured as a dichotomous variable coded “1” undergraduate and coded “0” for graduates
English proficiency	30 statements for which the respondents need to select the correct answer.
International experience	International experience was measured with three items (“Years of study abroad,” “Years of work abroad,” and “Total years of tourism abroad”) and was averaged across the three items
Work experience	Work experience was measured with the question “How many years of work experience do you have?” and seven response options ranging from 1 = “No work experience” to 7 = “More than 10 years”
Current employment	Current employment was measured with the question “Do you have a job at this time (in addition to your studies)?” and five response options ranging from 1 = “Full‐time student and do not work” to 5 = “I run a business full‐time with at least five full‐time employees”
Team size	Team size was measured based on the number of team members in a GVT
Team time zone difference	Team time zone difference was measured as the average inter‐member difference in hours between time zones among the team members
Team cultural distance	Team cultural distance was measured as the average cultural distance between team members using Kogut and Singh's (1988) index and the four core Hofstede (2001) dimensions.
Percent of course grade	“What percentage of your total course grade is accounted for by the project?”
Masculinity	Four survey items based on Yoo et al. (2011) with a 5‐point Likert scale anchored in 1 = *strongly disagree* and 5 = *strongly agree*. Example items are “Solving difficult problems usually requires an active forcible approach, which is typical for men,” “Men usually solve problems with logical analysis; women usually solve problems with intuition,” and “It is more important for men to have a professional career than it is for women.”
Power distance	Five survey items based on Yoo et al. (2011) with a 5‐point Likert scale anchored in 1 = *strongly disagree* and 5 = *strongly agree*. Example items are “People in higher positions should make most decisions without consulting people in lower positions,” “People in lower positions should not ask the opinions of people in lower positions,” and “People in higher positions should not delegate important task to people in lower positions.”
COVID‐19 impact on general life	Nine answers (“yes” = 1; “no” = 0) to the general question “Which of the following measures have been implemented to stop the spread of COVID‐19 and affected your life and work?” in association with the following general life aspects: (1) My university courses converted to online delivery. (2) Semester schedule altered (e.g., shorter semester). (3) University facilities closed (e.g., libraries and laboratories). (4) A requirement to vacate on‐campus dormitory. (5) City facilities closed (e.g., public libraries, gyms, stadiums, and parks). (6) Disrupted supply of food and other essentials (food stores closed and hard to buy other essentials). (7) Ban on leaving home except for essential travel and limited time (e.g., only to buy food and only close to home for a limited time). (8) Canceled travel plans, conferences, graduation ceremonies, and other events. (9) Complete quarantine
COVID‐19 impact on own job	Answer (“yes” = 1; “no” = 0) to the question “If you had a job before the crisis started, did the COVID‐19 affected your employment?”
COVID‐19 impact on parents' job	Answer (“yes” = 1; “no” = 0) to the question “If you depended on parents (or guardians) for support before the crisis started, did the COVID‐19 affected their employment?”

**TABLE A3:** Regression results for the full model and a model without controls

Variable and statistics	Individual performance						Self‐regulation					
	Model 1			Model 2			Model 3			Model 4		
	*B*	(*p*)	*SE*	*B*	(*p*)	*SE*	*B*	(*p*)	*SE*	*B*	(*p*)	*SE*
Controls												
Age	−.01	(.403)	.00				.01	(.058)	.00			
Gender (1 = female)	.13	(.000)***	.03				−.11	(.000)***	.01			
Study level (1 = undergraduate)	−.01	(.861)	.06				−.01	(.813)	.04			
English proficiency	.01	(.001)**	.00				.00	(.159)	.00			
Languages	.03	(.048)*	.02				.02	(.080)	.01			
International experience	−.02	(.006)**	.01				.02	(.157)	.01			
Work experience	.04	(.077)	.02				.01	(.192)	.01			
Employment	−.03	(.013)*	.01				−.01	(.465)	.01			
Team size	−.02	(.182)	.02				.01	(.819)	.01			
Team cultural distance	−.02	(.391)	.00				.01	(.230)	.00			
Team time zone difference	−.02	(.182)	.00				.01	(.819)	.00			
Percent of course grade	.01	(.560)	.09				.05	(.410)	.06			
Masculinity	−.05	(.036)*	.02				−.01	(.930)	.01			
Power distance	−.08	(.005)**	.02				−.07	(.000)***	.02			
Stringency index	.01	(.050)	.00				−.01	(.379)	.00			
GDP	.08	(.115)	.03				−.01	(.597)	.02			
COVID‐19 impact on general life	.01	(.146)	.01				.01	(.031)*	.01			
COVID‐19 impact on own job	−.01	(.229)	.01				−.01	(.213)	.00			
COVID‐19 impact on parents' job	−.01	(.002)**	.00				.00	(.653)	.00			
Direct effects												
Uncertainty avoidance	−.06	(.000)***	.02	−.04	(.228)	.03	.16	(.000)***	.01	.16	(.000)***	.02
Long‐term orientation	.08	(.000)***	.02	.10	(.000)***	.02	.28	(.000)***	.02	.30	(.000)***	.02
Collectivism	.02	(.219)	.02	−.02	(.280)	.02	.03	(.143)	.02	.04	(.101)	.02
Self‐regulation	.04	(.000)***	.03	.05	(.001)***	.01						
COVID‐19 (Δ deaths week in %)	−.07	(.000)*	.02	−.10	(.015)*	.04						
Moderation effect												
Self‐regulation × COVID‐19 (Δ deaths week in %)	.03	(.000)***	.01	.02	(.015)*	.01						
Intercept	3.30	(.000)***	.32	3.94	(.000)***	.12	1.96	(.000)***	.23	1.80	(.000)***	.13
*F*		142.68	(.000)***		9.70	(.000)***		308.05	(.000)***		91.87	(.000)***
*R* ^2^		.094			.035			.19			.171	
Adjusted *R* ^2^		.086			.032			.19			.171	

*Note*: *N* = 2727. Unstandardized estimates. Cluster‐robust standard errors for country (*N* = 31). Gender: 1 = female, 0 = male. Study level: 1 = undergraduate, 0 = graduate. Exact *p* values in parentheses.

* *p* < .05.

** *p* < .01.

*** *p* < .001.

#### A.1 Student samples in GVT research

Although the generalizability of findings based on student samples has been called into question (e.g., Bello et al., 2009; Shen et al., 2011), the main concerns about student samples only have limited relevance in the context of the present study (Hanel & Vione, 2016). The GVTs were asked to develop solutions to real‐life international business challenges presented by actual corporate clients. The work design of the project was closely reminiscent of actual business consulting projects, and the telework settings were the same as those experienced by corporate employees who were forced to work from home during the COVID‐19 pandemic (e.g., they were isolated in their homes, had weekly deadlines, and relied on online meetings and virtual communication tools to interact with their team members). The participants were concerned about and affected by the pandemic, as demonstrated by the analysis of the comments they had written in their weekly performance surveys (see Table 3). Given that participants in the GVTs were spread across the globe, the cultural diversity, geographic dispersion, and time zone differences were real and not simulated. Sometimes, this occurs in studies that rely on student convenience samples, as would be the case in actual corporate GVTs (Hoch & Kozlowski, 2014). Unlike most studies relying on student samples that offer students a small grade bonus for completing a survey or taking part in a simulation, the stakes were extremely high in the identified project. Depending on the university at which participants were enrolled, the project accounted for 20 to 100% of the course grade (36% on average). Failing the project meant failing the course. Additionally, most corporate clients offered after‐market commission and the possibility of internships or even job offers for the members of the best teams. Finally, only students who completed the project received project certificates and positive recommendation letters. While these incentives did not amount to or had the effect of a real salary, they were strong enough to motivate the students to genuinely do their best and to take the project very seriously.

#### A.2 Assessment of interrater reliability for peer‐rated individual performance

The main outcome variable of this study is individual performance measured by peer evaluation of five performance facets the GVT members assessed for each team member. Given that individual‐level responses of peers were aggregated to form this performance measure, within‐team interrater reliability in the form of multi‐item *r*_WG(*J*)_ was used to evaluate whether data aggregation was empirically justifiable (LeBreton & Senter, 2008). In this study, the size of each sample team was relatively small, and thus, multi‐item *r*_WG(*J*)_ was a better index to use as criteria for data aggregation than intraclass correlation coefficients. Using a format recommended by Biemann et al. (2012), in addition to reporting *r*_WG(*J*)_ with respect to the uniform null distribution of error (i.e., *r*_WG(*J*).uniform_), we also report *r*_WG(*J*).measure‐specific_ based on a slightly skewed null distribution of error. Since the GVT members might have had a bias whereby peers in GVTs could have evaluated the performance of their teammates favorably due to politeness, a slightly skewed null distribution of error for a measure with five response options was used for this computation (LeBreton & Senter, 2008). The mean *r*_WG(*J*).uniform_ was .85 (*SD* = 0.17), and the mean *r*_WG(*J*).measure‐specific_ was .72 (*SD* = 0.28). The median *r*_WG(*J*).uniform_ was .91, and the median *r*_WG(*J*).measure‐specific_ was .82. In sum, all *r*_WG(*J*)_ values are greater than .7, suggesting a good interrater reliability and the adequacy of the aggregation of peer evaluations to an individual performance measure (LeBreton & Senter, 2008).

#### A.3 Description of country‐level control variables and pandemic‐related survey items

We included several control variables related to the COVID‐19 pandemic to rule out alternative explanations (Becker et al., 2016). First, we used the *stringency index* of the Oxford COVID‐19 Government Response Tracker (OxCGRT) to account for the common policy responses introduced by governments in each of the 31 countries included in our sample. This score is a composite measure based on 20 indicators on a scale of 1 to 100. Previous research theoretically argues and empirically shows that governments' responses to the pandemic have influenced citizens mental well‐being (e.g., Fetzer et al., 2020). Higher stringency scores indicated a stricter pandemic response. Eight of the 20 indicators were related to containment and closure policies (e.g., movement restrictions). Four of the 20 indicators were related to economic policies (e.g., income support). Eight of the 20 indicators covered health system policies (e.g., the COVID‐19 testing regime). In addition to the overall stringency index, we also examined the three constituting facets, that is, the containment and health index (e.g., measures such as “lockdown” restrictions, testing policy, and contact tracing), the economic support index, and the government response index. We used stringency scores data for the same point in time as the COVID data (i.e., 5 days before we measured self‐regulation). Moreover, we also included the logarithm of countries' gross domestic product (GDP) values, represented in billions of US dollars, in the analyses. Previous research shows that economic development is associated with peers' evaluation of team members performance in GVTs (e.g., Tavoletti et al., 2022).

For the majority of countries included in our sample, secondary data is only available at the country level and not at a regional level. At the pandemic's onset differences in COVID‐19 severity not only appeared between countries but also between regions within a country. To account for these otherwise unobserved potential differences, we also included pandemic‐related variables based on our primary data. In addition to the secondary data sources, we used primary data from the survey to assess three specific aspects related to the pandemic: the perceived general impact of the pandemic on respondents' personal life (*general impact*), the perceived impact on respondents' jobs (*own job impact*), and the perceived impact on the jobs of parents of the respondents (*parents' job impact*). We measured general impact based on responses (“yes” = 1; “no” = 0) to nine questions on whether the pandemic had an impact on nine aspects of participants' lives (e.g., online courses, semester schedule, university facilities, campus dormitory, city facilities, food/essentials supply, travel restrictions, events, and quarantine). We summed the answers to the nine questions to calculate a total score, ranging from zero to nine, with higher scores representing a stronger general impact of the pandemic on participants' lives. We used single items to assess the influence of the pandemic on participants' jobs and the jobs of their parents or guardians, respectively. The items were assessed using a 7‐point Likert scale (1 = *no change* to 7 = *laid off*).

#### A.4 Assessment of measurement invariance and common method bias

We assessed measurement invariance (Vandenberg & Lance, 2000) for individual cultural value orientations and the self‐regulation measure. Given the small sample sizes for various countries in our sample (*n* < 200), we followed earlier studies (e.g., Gunkel et al., 2016) and examined measurement invariance across broad cultural clusters (Ronen & Shenkar, 2013). We used confirmatory factor analysis (CFA) and the comparative fit index (CFI; .9 or higher), as well as the root mean square error of approximation (RMSEA; below .08) to test the measurement model (Cheung & Rensvold, 2002). The CFA results for the different clusters showed a good fit. We tested the measurement invariance across clusters using a multi‐group confirmatory factor analysis (MGCFA). The MGCFA results showed configural and partial metric invariance, indicating that the samples from the different clusters could be combined in a single sample. While we collected data on individual cultural value orientations and self‐regulation at different points in time, these variables were self‐reported, which can lead to a potential common method bias (Podsakoff et al., 2003). We conducted an additional CFA with the pooled sample, to which we added a common latent factor to the measurement model (Podsakoff et al., 2003). The common latent factor loadings were insignificant for this model, suggesting that common method bias had no significant influence. The detailed results of these analyses are available upon request.

## References

[job2671-bib-0001] Adamovic, M. (2018). An employee‐focused human resource management perspective for the management of global virtual teams. The International Journal of Human Resource Management, 29(14), 2159–2187. 10.1080/09585192.2017.1323227

[job2671-bib-0002] Allen, T. D. , Golden, T. D. , & Shockley, K. M. (2015). How effective is telecommuting? Assessing the status of our scientific findings. Psychological Science in the Public Interest, 16(2), 40–68. 10.1177/1529100615593273 26403188

[job2671-bib-0003] Ayoko, O. B. , Konrad, A. M. , & Boyle, M. V. (2012). Online work: Managing conflict and emotions for performance in virtual teams. European Management Journal, 30(2), 156–174. 10.1016/j.emj.2011.10.001

[job2671-bib-0004] Bagozzi, R. P. (1992). The self‐regulation of attitudes, intentions, and behavior. Social Psychology Quarterly, 55(2), 178–204. 10.2307/2786945

[job2671-bib-0005] Bandura, A. (1988). Self‐regulation of motivation and action through goal systems. In Cognitive perspectives on emotion and motivation (pp. 37–61). Springer. 10.1007/978-94-009-2792-6_2

[job2671-bib-0006] Bandura, A. (1991). Social cognitive theory of self‐regulation. Organizational Behavior and Human Decision Processes, 50(2), 248–287. 10.1016/0749-5978(91)90022-L

[job2671-bib-0007] Bateman, T. S. , & Barry, B. (2012). Masters of the long haul: Pursuing long‐term work goals. Journal of Organizational Behavior, 33(7), 984–1006. 10.1002/job.1778

[job2671-bib-0008] Baumeister, R. F. , & Vohs, K. D. (2007). Self‐regulation, ego depletion, and motivation. Social and Personality Psychology Compass, 1(1), 115–128. 10.1111/j.1751-9004.2007.00001.x

[job2671-bib-0009] Beal, D. J. , Weiss, H. M. , Barros, E. , & MacDermid, S. M. (2005). An episodic process model of affective influences on performance. Journal of Applied Psychology, 90(6), 1054–1068. 10.1037/0021-9010.90.6.1054 16316265

[job2671-bib-0010] Bearden, W. O. , Money, R. B. , & Nevins, J. L. (2006). A measure of long‐term orientation: Development and validation. Journal of the Academy of Marketing Science, 34(3), 456–467. 10.1177/0092070306286706

[job2671-bib-0011] Becker, T. E. , Atinc, G. , Breaugh, J. A. , Carlson, K. D. , Edwards, J. R. , & Spector, P. E. (2016). Statistical control in correlational studies: 10 essential recommendations for organizational researchers. Journal of Organizational Behavior, 37(2), 157–167. 10.1002/job.2053

[job2671-bib-0012] Bello, D. , Leung, K. , Radebaugh, L. , Tung, R. L. , & van Witteloostuijn, A. (2009). From the editors: Student samples in international business research. Journal of International Business Studies, 40(3), 361–364. 10.1057/jibs.2008.101

[job2671-bib-0013] Biemann, T. , Cole, M. S. , & Voelpel, S. (2012). Within‐group agreement: On the use (and misuse) of *r* _ *WG* _ and r_ *WG*(*J*)_ in leadership research and some best practice guidelines. The Leadership Quarterly, 23(1), 66–80. 10.1016/j.leaqua.2011.11.006

[job2671-bib-0014] Blanchard, A. L. (2021). The effects of COVID‐19 on virtual working within online groups. Group Processes & Intergroup Relations, 24(2), 290–296. 10.1177/1368430220983446

[job2671-bib-0015] Boekaerts, M. , Maes, S. , & Karoly, P. (2005). Self‐regulation across domains of applied psychology: Is there an emerging consensus? Applied Psychology, 54(2), 149–154. 10.1111/j.1464-0597.2005.00201.x

[job2671-bib-0016] Bolino, M. C. , Harvey, J. , & Bachrach, D. G. (2012). A self‐regulation approach to understanding citizenship behavior in organizations. Organizational Behavior and Human Decision Processes, 119(1), 126–139. 10.1016/j.obhdp.2012.05.006

[job2671-bib-0017] Brown, J. M. , Miller, W. R. , & Lawendowski, L. A. (1999). The self‐regulation questionnaire. In L. VandeCreek & T. L. Jackson (Eds.), Innovations in clinical practice: A sourcebook. 17 (pp. 281–292). Professional Resource Press/Professional Resource Exchange.

[job2671-bib-0018] Burnette, J. L. , O'Boyle, E. H. , VanEpps, E. M. , Pollack, J. M. , & Finkel, E. J. (2013). Mind‐sets matter: A meta‐analytic review of implicit theories and self‐regulation. Psychological Bulletin, 139(3), 655–701. 10.1037/a0029531 22866678

[job2671-bib-0019] Caligiuri, P. , De Cieri, H. , Minbaeva, D. , Verbeke, A. , & Zimmermann, A. (2020). International HRM insights for navigating the COVID‐19 pandemic: Implications for future research and practice. Journal of International Business Studies, 51, 697–713. 10.1057/s41267-020-00335-9 32836500 PMC7266413

[job2671-bib-0020] Campbell, J. M. (1983). Ambient stressors. Environment and Behavior, 15(3), 355–380. 10.1177/0013916583153005

[job2671-bib-0021] Carver, C. S. , & Scheier, M. F. (1981). On the self‐regulation of behavior. Cambridge University Press. 10.1017/CBO9781139174794

[job2671-bib-0022] Carver, C. S. , & Scheier, M. F. (2000). On the structure of behavioral self‐regulation. In Handbook of self‐regulation (pp. 41–84). Academic Press. 10.1016/B978-012109890-2/50032-9

[job2671-bib-0163] Carver, C. S. , & Scheier, M. F. (2011). Self‐regulation of action and affect. In K. D. Vohs & R. F. Baumeister Handbook of self‐regulation: Research, theory, and applications (pp. 3–21). The Guilford Press.

[job2671-bib-0023] Cellar, D. F. , Stuhlmacher, A. F. , Young, S. K. , Fisher, D. M. , Adair, C. K. , Haynes, S. , Twichell, E. , Arnold, K. A. , Royer, K. , Denning, B. L. , & Riester, D. (2011). Trait goal orientation, self‐regulation, and performance: A meta‐analysis. Journal of Business and Psychology, 26(4), 467–483. 10.1007/s10869-010-9201-6

[job2671-bib-0024] Cheung, G. W. , & Rensvold, R. B. (2002). Evaluating goodness‐of‐fit indexes for testing measurement invariance. Structural Equation Modeling: A Multidisciplinary Journal, 9(2), 233–255. 10.1207/S15328007SEM0902_5

[job2671-bib-0025] Cho, E. (2020). Examining boundaries to understand the impact of COVID‐19 on vocational behaviors. Journal of Vocational Behavior, 119, 103437. 10.1016/j.jvb.2020.103437 32390657 PMC7205716

[job2671-bib-0166] Clugston, M. , Howell, J. P. , & Dorfman, P. W. (2000). Does cultural socialization predict multiple bases and foci of commitment? Journal of Management, 26(1), 5–30.

[job2671-bib-0026] Cogliser, C. C. , Gardner, W. L. , Gavin, M. B. , & Broberg, J. C. (2012). Big five personality factors and leader emergence in virtual teams: Relationships with team trustworthiness, member performance contributions, and team performance. Group & Organization Management, 37(6), 752–784. 10.1177/1059601112464266

[job2671-bib-0027] Collings, D. G. , McMackin, J. , Nyberg, A. J. , & Wright, P. M. (2021). Strategic human resource management and COVID‐19: Emerging challenges and research opportunities. Journal of Management Studies, 58, 1378–1382. 10.1111/joms.12695

[job2671-bib-0028] Connaughton, S. L. , & Shuffler, M. (2007). Multinational and multicultural distributed teams: A review and future agenda. Small Group Research, 38(3), 387–412. 10.1177/1046496407301970

[job2671-bib-0029] Conway, J. M. (1999). Distinguishing contextual performance from task performance for managerial jobs. Journal of Applied Psychology, 84(1), 3–13. 10.1037/0021-9010.84.1.3

[job2671-bib-0030] Cortina, J. M. , & Landis, R. S. (2009). When small effect sizes tell a big story, and when large effect sizes don't. In C. E. Lance & R. J. Vandenberg (Eds.), Statistical and methodological myths and urban legends: Doctrine, verity and fable in the organizational and social sciences (pp. 287–308). Routledge.

[job2671-bib-0031] Dawson, J. F. (2014). Moderation in management research: What, why, when and how. Journal of Business and Psychology, 29(1), 1–19. 10.1007/s10869-013-9308-7

[job2671-bib-0032] de Jonge, J. , & Dormann, C. (2006). Stressors, resources, and strain at work: A longitudinal test of the triple‐match principle. Journal of Applied Psychology, 91(6), 1359–1374. 10.1037/0021-9010.91.5.1359 17100490

[job2671-bib-0033] Degbey, W. Y. , & Einola, K. (2020). Resilience in virtual teams: Developing the capacity to bounce back. Applied Psychology, 69(4), 1301–1337. 10.1111/apps.12220

[job2671-bib-0034] Dekker, D. M. , Rutte, C. G. , & Van den Berg, P. T. (2008). Cultural differences in the perception of critical interaction behaviors in global virtual teams. International Journal of Intercultural Relations, 32(5), 441–452. 10.1016/j.ijintrel.2008.06.003

[job2671-bib-0165] Dong, E. , Du, H. , & Gardner, L. (2020). An interactive web‐based dashboard to track COVID‐19 in real time. The Lancet Infectious Diseases, 20(5), 533–534. 32087114 10.1016/S1473-3099(20)30120-1 PMC7159018

[job2671-bib-0035] Evans, G. W. (1984). Environmental stress. University of California.

[job2671-bib-0036] Farh, J. L. , Hackett, R. D. , & Liang, J. (2007). Individual‐level cultural values as moderators of perceived organizational support–employee outcome relationships in China: Comparing the effects of power distance and traditionality. Academy of Management Journal, 50(3), 715–729. 10.5465/amj.2007.25530866

[job2671-bib-0037] Felstead, A. , Jewson, N. , & Walters, S. (2003). Managerial control of employees working at home. British Journal of Industrial Relations, 41(2), 241–264. 10.1111/1467-8543.00271

[job2671-bib-0038] Fetzer, T. , Witte, M. , Hensel, L. , Jachimowicz, J. , Haushofer, J. , Ivchenko, A. , Caria, S. , Reutskaja, E. , Roth, C. , Fiorin, S. , Gómez, M. , Kraft‐Todd, G. , Götz, F. M. , & Yoeli, E. (2020). Perceptions of an insufficient government response at the onset of the COVID‐19 pandemic are associated with lower mental well‐being. 10.31234/osf.io/3kfmh

[job2671-bib-0040] Forester, G. L. , Thorns, P. , & Pinto, J. K. (2007). Importance of goal setting in virtual project teams. Psychological Reports, 100(1), 270–274. 10.2466/pr0.100.1.270-274 17451035

[job2671-bib-0041] Fu, S. , Greco, L. M. , Lennard, A. C. , & Dimotakis, N. (2021). Anxiety responses to the unfolding COVID‐19 crisis: Patterns of change in the experience of prolonged exposure to stressors. Journal of Applied Psychology, 106(1), 48–61. 10.1037/apl0000855 33271021

[job2671-bib-0042] Garfin, D. R. , Silver, R. C. , & Holman, E. A. (2020). The novel coronavirus (COVID‐2019) outbreak: Amplification of public health consequences by media exposure. Health Psychology, 39(5), 355–357. 10.1037/hea0000875 32202824 PMC7735659

[job2671-bib-0043] Gilboa, S. , Shirom, A. , Fried, Y. , & Cooper, C. (2008). A meta‐analysis of work demand stressors and job performance: Examining main and moderating effects. Personnel Psychology, 61(2), 227–271. 10.1111/j.1744-6570.2008.00113.x

[job2671-bib-0044] Glazer, S. , Kożusznik, M. W. , & Shargo, I. A. (2012). Global virtual teams: A cure for–or a cause of–stress. In The role of the economic crisis on occupational stress and well‐being. Emerald Group Publishing Limited. 10.1108/S1479-3555(2012)0000010010

[job2671-bib-0045] Götz, F. M. , Gosling, S. D. , & Rentfrow, P. J. (2022). Small effects: The indispensable foundation for a cumulative psychological science. Perspectives on Psychological Science, 17(1), 205–215. 10.1177/174569162098448 34213378

[job2671-bib-0046] Gross, J. J. , & Thompson, R. A. (2007). Emotion regulation: Conceptual foundations. In J. J. Gross (Ed.), Handbook of emotion regulation (pp. 3–24). The Guilford Press.

[job2671-bib-0047] Guan, Y. , Deng, H. , & Zhou, X. (2020). Understanding the impact of the COVID‐19 pandemic on career development: Insights from cultural psychology. Journal of Vocational Behavior, 119, 103438. 10.1016/j.jvb.2020.103438 32382162 PMC7204647

[job2671-bib-0048] Gunkel, M. , Schlaegel, C. , & Taras, V. (2016). Cultural values, emotional intelligence, and conflict handling styles: A global study. Journal of World Business, 51(4), 568–585. 10.1016/j.jwb.2016.02.001

[job2671-bib-0049] Halbesleben, J. R. , & Bowler, W. M. (2007). Emotional exhaustion and job performance: The mediating role of motivation. Journal of Applied Psychology, 92(1), 93–106. 10.1037/0021-9010.92.1.93 17227154

[job2671-bib-0050] Hamilton, R. , Vohs, K. D. , Sellier, A. L. , & Meyvis, T. (2011). Being of two minds: Switching mindsets exhausts self‐regulatory resources. Organizational Behavior and Human Decision Processes, 115(1), 13–24. 10.1016/j.obhdp.2010.11.005

[job2671-bib-0051] Hanel, P. H. P. , & Vione, K. C. (2016). Do student samples provide an accurate estimate of the general public? PLoS ONE, 11(12), e0168354. 10.1371/journal.pone.0168354 28002494 PMC5176168

[job2671-bib-0052] Hanna, A. A. , Smith, T. A. , Kirkman, B. L. , & Griffin, R. W. (2021). The emergence of emergent leadership: A comprehensive framework and directions for future research. Journal of Management, 47(1), 76–104. 10.1177/0149206320965683

[job2671-bib-0053] Hardin, A. M. , Fuller, M. A. , & Davison, R. M. (2007). I know I can, but can we? Culture and efficacy beliefs in global virtual teams. Small Group Research, 38(1), 130–155. 10.1177/1046496406297041

[job2671-bib-0055] Harzing, A. W. (2005). Does the use of English‐language questionnaires in cross‐national research obscure national differences? International Journal of Cross Cultural Management, 5(2), 213–224. 10.1177/1470595805054494

[job2671-bib-0056] Helton, W. S. , & Head, J. (2012). Earthquakes on the mind: Implications of disasters for human performance. Human Factors, 54(2), 189–194. 10.1177/0018720811430503 22624286

[job2671-bib-0057] Hertel, G. , Geister, S. , & Konradt, U. (2005). Managing virtual teams: A review of current empirical research. Human Resource Management Review, 15(1), 69–95. 10.1016/j.hrmr.2005.01.002

[job2671-bib-0058] Hobfoll, S. E. (1988). The ecology of stress. Hemisphere.

[job2671-bib-0059] Hobfoll, S. E. (1989). Conservation of resources: A new attempt at conceptualizing stress. American Psychologist, 44(3), 513–524. 10.1037//0003-066x.44.3.513 2648906

[job2671-bib-0060] Hobfoll, S. E. (2001). The influence of culture, community, and the nested‐self in the stress process: Advancing conservation of resources theory. Applied Psychology, 50(3), 337–421. 10.1111/1464-0597.00062

[job2671-bib-0061] Hobfoll, S. E. (2002). Social and psychological resources and adaptation. Review of General Psychology, 6(4), 307–324. 10.1037/1089-2680.6.4.307

[job2671-bib-0062] Hobfoll, S. E. (2011). Conservation of resource caravans and engaged settings. Journal of Occupational and Organizational Psychology, 84(1), 116–122. 10.1111/j.2044-8325.2010.02016.x

[job2671-bib-0063] Hobfoll, S. E. (2012). Conservation of resources and disaster in cultural context: The caravans and passageways for resources. Psychiatry, 75(3), 227–232. 10.1521/psyc.2012.75.3.227 22913498

[job2671-bib-0064] Hobfoll, S. E. , Halbesleben, J. , Neveu, J. P. , & Westman, M. (2018). Conservation of resources in the organizational context: The reality of resources and their consequences. Annual Review of Organizational Psychology and Organizational Behavior, 5(1), 103–128. 10.1146/annurev-orgpsych-032117-104640

[job2671-bib-0067] Hoch, J. E. , & Kozlowski, S. W. (2014). Leading virtual teams: Hierarchical leadership, structural supports, and shared team leadership. Journal of Applied Psychology, 99(3), 390–403. 10.1037/a0030264 23205494

[job2671-bib-0068] Hofstede, G. (1984). Culture's consequences: International differences in work‐related values. Sage.

[job2671-bib-0069] Hofstede, G. (2001). Culture's consequences: Comparing values, behaviors, institutions, and organizations across nations (2nd ed.). Sage Publications.

[job2671-bib-0070] House, R. , Javidan, M. , Hanges, P. , & Dorfman, P. (2002). Understanding cultures and implicit leadership theories across the globe: An introduction to project GLOBE. Journal of World Business, 37(1), 3–10. 10.1016/S1090-9516(01)00069-4

[job2671-bib-0071] Hu, J. , He, W. , & Zhou, K. (2020). The mind, the heart, and the leader in times of crisis: How and when COVID‐19‐triggered mortality salience relates to state anxiety, job engagement, and prosocial behavior. Journal of Applied Psychology, 105(11), 1218–1233. 10.1037/apl0000620 33030924

[job2671-bib-0072] Hui, C. H. , & Triandis, H. C. (1986). Individualism‐collectivism: A study of cross‐cultural researchers. Journal of Cross‐Cultural Psychology, 17(2), 225–248. 10.1177/0022002186017002006

[job2671-bib-0073] Hunter, L. W. , & Thatcher, S. M. B. (2007). Feeling the heat: Effects of stress, commitment, and job experience on job performance. Academy of Management Journal, 50(4), 953–968. 10.5465/amj.2007.26279227

[job2671-bib-0076] Jimenez, A. , Boehe, D. M. , Taras, V. , & Caprar, D. V. (2017). Working across boundaries: Current and future perspectives on global virtual teams. Journal of International Management, 23(4), 341–349. 10.1016/j.intman.2017.05.001

[job2671-bib-0077] Kanfer, R. (2005). Self‐regulation research in work and I/O psychology. Applied Psychology, 54(2), 186–191. 10.1111/j.1464-0597.2005.00203.x

[job2671-bib-0078] Kanfer, R. , & Ackerman, P. L. (1989). Motivation and cognitive abilities: An integrative/aptitude‐treatment interaction approach to skill acquisition. Journal of Applied Psychology, 74(4), 657–690. 10.1037/0021-9010.74.4.657

[job2671-bib-0079] Keith, N. , & Frese, M. (2005). Self‐regulation in error management training: Emotion control and metacognition as mediators of performance effects. Journal of Applied Psychology, 90(4), 677–691. 10.1037/0021-9010.90.4.677 16060786

[job2671-bib-0080] Kitayama, S. (2000). Collective construction of the self and social relationships: A rejoinder and some extensions. Child Development, 71(5), 1143–1146. 10.1111/1467-8624.00215 11108083

[job2671-bib-0082] Klonek, F. E. , Kanse, L. , Wee, S. , Runneboom, C. , & Parker, S. K. (2021). Did the COVID‐19 lock‐down make us better at working in virtual teams? Small Group Research., 53, 185–206. 10.1177/10464964211008991

[job2671-bib-0083] Kniffin, K. M. , Narayanan, J. , Anseel, F. , Antonakis, J. , Ashford, S. P. , Bakker, A. B. , … Vugt, M. V. (2021). COVID‐19 and the workplace: Implications, issues, and insights for future research and action. American Psychologist, 76(1), 63–77. 10.1037/amp0000716 32772537

[job2671-bib-0084] Kogut, B. , & Singh, H. (1988). The effect of national culture on the choice of entry mode. Journal of International Business Studies, 19(3), 411–432. 10.1057/palgrave.jibs.8490394

[job2671-bib-0085] Kowal, M. , Coll‐Martín, T. , Ikizer, G. , Rasmussen, J. , Eichel, K. , Studzińska, A. , Koszałkowska, K. , Karwowski, M. , Najmussaqib, A. , Pankowski, D. , Lieberoth, A. , & Ahmed, O. (2020). Who is the most stressed during the COVID‐19 pandemic? Data from 26 countries and areas. Applied Psychology: Health and Well‐Being, 12(4), 946–966. 10.1111/aphw.12234 32996217 PMC7537225

[job2671-bib-0086] Kumar, R. , Budhwar, P. , Patel, C. , & Varma, A. (2019). Self‐regulation and expatriate adjustment: The role of regulatory fit. Human Resource Management Review, 29(4), 100666. 10.1016/j.hrmr.2018.09.002

[job2671-bib-0087] Kuntz, J. R. C. , Näswall, K. , & Malinen, S. (2016). Resilient employees in resilient organizations: Flourishing beyond adversity. Industrial and Organizational Psychology, 9(2), 456–462. 10.1017/iop.2016.39

[job2671-bib-0088] Kurman, J. (2001). Self‐regulation strategies in achievement settings: Culture and gender differences. Journal of Cross‐Cultural Psychology, 32(4), 491–503. 10.1177/0022022101032004008

[job2671-bib-0090] LeBreton, J. M. , & Senter, J. L. (2008). Answers to 20 questions about interrater reliability and interrater agreement. Organizational Research Methods, 11(4), 815–852. 10.1177/1094428106296642

[job2671-bib-0091] LePine, J. A. , Podsakoff, N. P. , & LePine, M. A. (2005). A meta‐analytic test of the challenge stressor–hindrance stressor framework: An explanation for inconsistent relationships among stressors and performance. Academy of Management Journal, 48(5), 764–775. 10.5465/amj.2005.18803921

[job2671-bib-0092] Leung, K. (1989). Cross‐cultural differences: Individual‐level vs. culture‐level analysis. International Journal of Psychology, 24(6), 703–719. 10.1080/00207598908247840

[job2671-bib-0093] Lian, H. , Yam, K. C. , Ferris, D. L. , & Brown, D. (2017). Self‐control at work. Academy of Management Annals, 11(2), 703–732. 10.5465/annals.2015.0126

[job2671-bib-0095] Martins, L. L. , Gilson, L. L. , & Maynard, M. T. (2004). Virtual teams: What do we know and where do we go from here? Journal of Management, 30(6), 805–835. 10.1016/j.jm.2004.05.002

[job2671-bib-0096] Matsumoto, D. , Yoo, S. H. , Nakagawa, S. , & 37 members of the Multinational Study of Cultural Display Rules . (2008). Culture, emotion regulation, and adjustment. Journal of Personality and Social Psychology, 94(6), 925–937. 10.1037/0022-3514.94.6.925 18505309

[job2671-bib-0097] Matthews, S. H. , Kelemen, T. K. , & Bolino, M. C. (2021). How follower traits and cultural values influence the effects of leadership. The Leadership Quarterly, 32(1), 101497. 10.1016/j.leaqua.2021.101497

[job2671-bib-0098] Maynard, M. T. , Mathieu, J. E. , Rapp, T. L. , & Gilson, L. L. (2012). Something(s) old and something(s) new: Modeling drivers of global virtual team effectiveness. Journal of Organizational Behavior, 33(3), 342–365. 10.1002/job.1772

[job2671-bib-0100] McCarthy, J. M. , Erdogan, B. , & Bauer, T. N. (2019). An interpersonal perspective of perceived stress: Examining the prosocial coping response patterns of stressed managers. Journal of Organizational Behavior, 40(9–10), 1027–1044. 10.1002/job.2406

[job2671-bib-0101] McClelland, M. M. , & Cameron, C. E. (2012). Self‐regulation in early childhood: Improving conceptual clarity and developing ecologically valid measures. Child Development Perspectives, 6(2), 136–142. 10.1111/j.1750-8606.2011.00191.x

[job2671-bib-0102] McDaniel, S. H. , & Salas, E. (2018). The science of teamwork: Introduction to the special issue. American Psychologist, 73(4), 305–307. 10.1037/amp0000337 29792449

[job2671-bib-0103] McEwen, K. (2011). Building resilience at work. Australian Academic Press.

[job2671-bib-0104] McNeish, D. , Stapleton, L. M. , & Silverman, R. D. (2017). On the unnecessary ubiquity of hierarchical linear modeling. Psychological Methods, 22(1), 114–140. 10.1037/met0000078 27149401

[job2671-bib-0105] Mesmer‐Magnus, J. R. , DeChurch, L. A. , Jimenez‐Rodriguez, M. , Wildman, J. , & Shuffler, M. (2011). A meta‐analytic investigation of virtuality and information sharing in teams. Organizational Behavior and Human Decision Processes, 115(2), 214–225. 10.1016/j.obhdp.2011.03.002

[job2671-bib-0106] Mitchell, M. S. , Greenbaum, R. L. , Vogel, R. M. , Mawritz, M. B. , & Keating, D. J. (2019). Can you handle the pressure? The effect of performance pressure on stress appraisals, self‐regulation, and behavior. Academy of Management Journal, 62(2), 531–552. 10.5465/amj.2016.0646

[job2671-bib-0108] Monnier, J. , & Hobfoll, S. E. (2000). Conservation of resources in individual and community reactions to traumatic stress. In International handbook of human response to trauma (pp. 325–336). Springer. 10.1007/978-1-4615-4177-6_23

[job2671-bib-0110] Morelli, N. A. , & Cunningham, C. J. L. (2012). Not all resources are created equal: COR theory, values, and stress. Journal of Psychology, 146(4), 393–415. 10.1080/00223980.2011.650734 22808687

[job2671-bib-0111] Müller, T. , & Niessen, C. (2019). Self‐leadership in the context of part‐time teleworking. Journal of Organizational Behavior, 40(8), 883–898. 10.1002/job.2371

[job2671-bib-0112] Ng, T. W. , & Feldman, D. C. (2008). The relationship of age to ten dimensions of job performance. Journal of Applied Psychology, 93(2), 392–423. 10.1037/0021-9010.93.2.392 18361640

[job2671-bib-0113] Niessen, C. , & Jimmieson, N. L. (2016). Threat of resource loss: The role of self‐regulation in adaptive task performance. Journal of Applied Psychology, 101(3), 450–462. 10.1037/apl0000049 26348477

[job2671-bib-0114] Norris, F. H. , & Uhl, G. A. (1993). Chronic stress as a mediator of acute stress: The case of Hurricane Hugo 1. Journal of Applied Social Psychology, 23(16), 1263–1284. 10.1111/j.1559-1816.1993.tb01032.x

[job2671-bib-0115] Nurmi, N. (2011). Coping with coping strategies: How distributed teams and their members deal with the stress of distance, time zones and culture. Stress and Health, 27(2), 123–143. 10.1002/smi.1327 27486615

[job2671-bib-0116] Ollier‐Malaterre, A. (2010). Contributions of work—Life and resilience initiatives to the individual/organization relationship. Human Relations, 63(1), 41–62. 10.1177/0018726709342458

[job2671-bib-0117] Oyserman, D. , & Lee, S. W. S. (2007). Priming ‘culture’: Culture as situated cognition. In S. Kitayama & D. Cohen (Eds.), Handbook of cultural psychology (pp. 255–279). Guilford Press.

[job2671-bib-0118] Paterson, T. A. , Harms, P. D. , Steel, P. , & Credé, M. (2016). An assessment of the magnitude of effect sizes: Evidence from 30 years of meta‐analysis in management. Journal of Leadership & Organizational Studies, 23(1), 66–81. 10.1177/1548051815614321

[job2671-bib-0119] Podsakoff, P. M. , MacKenzie, S. B. , Lee, J. Y. , & Podsakoff, N. P. (2003). Common method biases in behavioral research: A critical review of the literature and recommended remedies. Journal of Applied Psychology, 88(5), 879–903. 10.1037/0021-9010.88.5.879 14516251

[job2671-bib-0120] Porath, C. L. , & Bateman, T. S. (2006). Self‐regulation: From goal orientation to job performance. Journal of Applied Psychology, 91(1), 185–192. 10.1037/0021-9010.91.1.185 16435948

[job2671-bib-0122] Presbitero, A. (2020). Foreign language skill, anxiety, cultural intelligence and individual task performance in global virtual teams: A cognitive perspective. Journal of International Management, 26(2), 100729. 10.1016/j.intman.2019.100729

[job2671-bib-0123] Restubog, S. L. D. , Ocampo, A. C. G. , & Wang, L. (2020). Taking control amidst the chaos: Emotion regulation during the COVID‐19 pandemic. Journal of Vocational Behavior, 119, 103440. 10.1016/j.jvb.2020.103440 32390659 PMC7206430

[job2671-bib-0124] Ronen, S. , & Shenkar, O. (2013). Mapping world cultures: Cluster formation, sources and implycations. Journal of International Business Studies, 44(9), 867–897. 10.1057/jibs.2013.42

[job2671-bib-0125] Roney, C. J. R. , & Sorrentino, R. M. (1995). Reducing self‐discrepancies or maintaining self‐congruence? Uncertainty orientation, self‐regulation, and performance. Journal of Personality and Social Psychology, 68(3), 485–497. 10.1037/0022-3514.68.3.485

[job2671-bib-0126] Roth, P. L. , Purvis, K. L. , & Bobko, P. (2012). A meta‐analysis of gender group differences for measures of job performance in field studies. Journal of Management, 38(2), 719–739. 10.1177/0149206310374774

[job2671-bib-0128] Salas, E. , Kozlowski, S. W. , & Chen, G. (2017). A century of progress in industrial and organizational psychology: Discoveries and the next century. Journal of Applied Psychology, 102(3), 589–598. 10.1037/apl0000206 28206773

[job2671-bib-0130] Shachaf, P. (2008). Cultural diversity and information and communication technology impacts on global virtual teams: An exploratory study. Information & Management, 45(2), 131–142. 10.1016/j.im.2007.12.003

[job2671-bib-0131] Shen, W. , Kiger, T. B. , Davies, S. E. , Rasch, R. L. , Simon, K. M. , & Ones, D. S. (2011). Samples in applied psychology: Over a decade of research in review. Journal of Applied Psychology, 96(5), 1055–1064. 10.1037/a0023322 21463013

[job2671-bib-0132] Shuper, P. A. , Sorrentino, R. M. , Otsubo, Y. , Hodson, G. , & Walker, A. M. (2004). A theory of uncertainty orientation: Implications for the study of individual differences within and across cultures. Journal of Cross‐Cultural Psychology, 35(4), 460–480. 10.1177/0022022104266109

[job2671-bib-0133] Sorrentino, R. M. , Smithson, M. , Hodson, G. , Roney, C. J. R. , & Marie Walker, A. M. (2003). The theory of uncertainty orientation: A mathematical reformulation. Journal of Mathematical Psychology, 47(2), 132–149. 10.1016/S0022-2496(02)00032-9

[job2671-bib-0134] Staples, D. S. , & Zhao, L. (2006). The effects of cultural diversity in virtual teams versus face‐to‐face teams. Group Decision and Negotiation, 15(4), 389–406. 10.1007/s10726-006-9042-x

[job2671-bib-0135] Stokes, P. , Smith, S. , Wall, T. , Moore, N. , Rowland, C. , Ward, T. , & Cronshaw, S. (2019). Resilience and the (micro‐)dynamics of organizational ambidexterity: Implications for strategic HRM. The International Journal of Human Resource Management, 30(8), 1287–1322. 10.1080/09585192.2018.1474939

[job2671-bib-0136] Stokols, D. (1982). Environmental psychology: A coming of age. In A. G. Kraut (Ed.), The G. Stanley Hall lecture series. The G. Stanley Hall lecture series (Vol. 2, pp. 159–205). American Psychological Association. 10.1037/10087-004

[job2671-bib-0137] Taras, V. , Baack, D. , Caprar, D. , Dow, D. , Froese, F. , Jimenez, A. , & Magnusson, P. (2019). Diverse effects of diversity: Disaggregating effects of diversity in global virtual teams. Journal of International Management, 25(4), 100689. 10.1016/j.intman.2019.100689

[job2671-bib-0138] Taras, V. , Gunkel, M. , Assouad, A. , Tavoletti, E. , Kraemer, J. , Jiménez, A. , Svirina, A. , Lei, W. S. , & Shah, G. (2021). The predictive power of university pedigree on the graduate's performance in global virtual teams. European Journal of International Management, 16(4), 555–584. 10.1504/EJIM.2021.118582

[job2671-bib-0139] Taras, V. , Kirkman, B. L. , & Steel, P. (2010). Examining the impact of culture's consequences: A three‐decade, multilevel, meta‐analytic review of Hofstede's cultural value dimensions. Journal of Applied Psychology, 95(3), 405–439. 10.1037/a0018938 20476824

[job2671-bib-0140] Tavoletti, E. , Stephens, R. D. , Taras, V. , & Dong, L. (2022). Nationality biases in peer evaluations: The country‐of‐origin effect in global virtual teams. International Business Review, 31(2), 101969. 10.1016/j.ibusrev.2021.101969

[job2671-bib-0141] Taylor, S. (2019). The psychology of pandemics: Preparing for the next global outbreak of infectious disease. Cambridge Scholars Publishing.

[job2671-bib-0142] Taylor, S. , Landry, C. A. , Paluszek, M. M. , Fergus, T. A. , McKay, D. , & Asmundson, G. J. G. (2020). Development and initial validation of the COVID Stress Scales. Journal of Anxiety Disorders, 72, 102232. 10.1016/j.janxdis.2020.102232 32408047 PMC7198206

[job2671-bib-0143] Triandis, H. C. (1995). Individualism and collectivism. Westview Press.

[job2671-bib-0144] Trommsdorff, G. (2009). Culture and development of self‐regulation. Social and Personality Psychology Compass, 3(5), 687–701. 10.1111/j.1751-9004.2009.00209.x

[job2671-bib-0146] Trommsdorff, G. , & Cole, P. M. (2011). Emotion, self‐regulation, and social behavior in cultural contexts. In X. C. K. H. Rubin (Ed.), Socio‐ emotional development in cultural context (pp. 131–163). Guilford Press.

[job2671-bib-0147] Trommsdorff, G. , & Rothbaum, F. (2008). Development of emotion regulation in cultural context. In M. Vandekerckhove , C. V. Scheve , S. Ismer , S. Jung , & S. Kronast (Eds.), Regulating emotions: Culture, social necessity, and biological inheritance (pp. 85–120). Blackwell. 10.1002/9781444301786.ch4

[job2671-bib-0149] Tsui, A. S. , & Ashford, S. J. (1994). Adaptive self‐regulation: A process view of managerial effectiveness. Journal of Management, 20(1), 93–121. 10.1177/014920639402000105

[job2671-bib-0150] Vancouver, J. B. (2008). Integrating self‐regulation theories of work motivation into a dynamic process theory. Human Resource Management Review, 18(1), 1–18. 10.1016/j.hrmr.2008.02.001

[job2671-bib-0151] Vancouver, J. B. , & Day, D. V. (2005). Industrial and organisation research on self‐regulation: From constructs to applications. Applied Psychology, 54(2), 155–185. 10.1111/j.1464-0597.2005.00202.x

[job2671-bib-0152] Vandenberg, R. J. , & Lance, C. E. (2000). A review and synthesis of the measurement invariance literature: Suggestions, practices, and recommendations for organizational research. Organizational Research Methods, 3(1), 4–70. 10.1177/109442810031002

[job2671-bib-0164] Vohs, K. D. , & Baumeister, R. F. (2004). Understanding self‐regulation. In R. F. Baumeister & K. D. Vohs Handbook of self‐regulation (pp. 1–12). Guilford.

[job2671-bib-0153] Wang, B. , Liu, Y. , Qian, J. , & Parker, S. K. (2020). Achieving effective remote working during the COVID‐19 pandemic: A work design perspective. Applied Psychology, 70(1), 16–59. 10.1111/apps.12290 33230359 PMC7675760

[job2671-bib-0154] Whillans, A. , Perlow, L. , & Turek, A. (2021). Experimenting during the shift to virtual team work: Learnings from how teams adapted their activities during the COVID‐19 pandemic. Information and Organization, 31(1), 100343. 10.1016/j.infoandorg.2021.100343

[job2671-bib-0155] Wood, R. (2005). New frontiers for self‐regulation research in IO psychology. Applied Psychology, 54(2), 192–198.

[job2671-bib-0156] World Economic Forum . (2020). Working from home was a luxury for the relatively affluent before coronavirus—Not any more. https://www.weforum.org/agenda/2020/03/working-from-home-coronavirus-workers-future-of-work/

[job2671-bib-0157] Wright, R. R. , Mohr, C. D. , Sinclair, R. R. , & Yang, L. Q. (2015). Sometimes less is more: Directed coping with interpersonal stressors at work. Journal of Organizational Behavior, 36(6), 786–805. 10.1002/job.2002

[job2671-bib-0158] Yeo, G. B. , & Frederiks, E. R. (2011). Cognitive and affective regulation: Scale validation and nomological network analysis. Applied Psychology, 60(4), 546–575. 10.1111/j.1464-0597.2011.00447.x

[job2671-bib-0159] Yi‐Feng Chen, N. , Crant, J. M. , Wang, N. , Kou, Y. , Qin, Y. , Yu, J. , & Sun, R. (2021). When there is a will there is a way: The role of proactive personality in combating COVID‐19. Journal of Applied Psychology, 106(2), 199–213. 10.1037/apl0000865 33600195

[job2671-bib-0160] Yoo, B. , Donthu, N. , & Lenartowicz, T. (2011). Measuring Hofstede's five dimensions of cultural values at the individual level: Development and validation of CVSCALE. Journal of International Consumer Marketing, 23(3–4), 193–210. 10.1080/08961530.2011.578059

